# Targeting of H19/cell adhesion molecules circuitry by GSK-J4 epidrug inhibits metastatic progression in prostate cancer

**DOI:** 10.1186/s12935-024-03231-6

**Published:** 2024-02-05

**Authors:** Valeria Pecci, Fabiola Troisi, Aurora Aiello, Sara De Martino, Angela Carlino, Vincenzo Fiorentino, Cristian Ripoli, Dante Rotili, Francesco Pierconti, Maurizio Martini, Manuela Porru, Francesco Pinto, Antonello Mai, Pier Francesco Bassi, Claudio Grassi, Carlo Gaetano, Alfredo Pontecorvi, Lidia Strigari, Antonella Farsetti, Simona Nanni

**Affiliations:** 1 Department of Translational Medicine and Surgery, Università Cattolica del Sacro Cuore, Largo F. Vito 1, Rome, 00168 Italy; 2 National Research Council (CNR)-IASI, Rome, Italy; 3 Fondazione “Policlinico Universitario A. Gemelli IRCCS”, Rome, Italy; 4 Department of Neuroscience, Università Cattolica del Sacro Cuore, Rome, Italy; 5 Dipartimento di Chimica e Tecnologie del Farmaco, Sapienza Università di Roma, Rome, Italy; 6 Department of Woman, Child and Public Health, Università Cattolica del Sacro Cuore, Rome, Italy; 7 Translational Oncology Research Unit, IRCCS- Regina Elena National Cancer Institute, Rome, Italy; 8 Laboratory of Epigenetics, Istituti Clinici Scientifici Maugeri IRCCS, Pavia, Italy; 9 Department of Medical Physics, S. Orsola, Malpighi University Hospital, Bologna, Italy

**Keywords:** lncRNA, Metastasis, Lysine demethylase, Preclinical models

## Abstract

**Background:**

About 30% of Prostate cancer (PCa) patients progress to metastatic PCa that remains largely incurable. This evidence underlines the need for the development of innovative therapies. In this direction, the potential research focus might be on long non-coding RNAs (lncRNAs) like H19, which serve critical biological functions and show significant dysregulation in cancer. Previously, we showed a transcriptional down-regulation of H19 under combined pro-tumoral estrogen and hypoxia treatment in PCa cells that, in turn, induced both E-cadherin and β4 integrin expression. H19, indeed, acts as transcriptional repressor of cell adhesion molecules affecting the PCa metastatic properties. Here, we investigated the role of H19/cell adhesion molecules circuitry on in vivo PCa experimental tumor growth and metastatic dissemination models.

**Methods:**

H19 was silenced in luciferase-positive PC-3 and 22Rv1 cells and in vitro effect was evaluated by gene expression, proliferation and invasion assays before and after treatment with the histone lysine demethylase inhibitor, GSK-J4. In vivo tumor growth and metastasis dissemination, in the presence or absence of GSK-J4, were analyzed in two models of human tumor in immunodeficient mice by in vivo bioluminescent imaging and immunohistochemistry (IHC) on explanted tissues. Organotypic Slice Cultures (OSCs) from fresh PCa-explant were used as ex vivo model to test GSK-J4 effects.

**Results:**

H19 silencing in both PC-3 and 22Rv1 cells increased: *i*) E-cadherin and β4 integrin expression as well as proliferation and invasion, *ii*) in vivo tumor growth, and *iii*) metastasis formation at bone, lung, and liver. Of note, treatment with GSK-J4 reduced lesions. In parallel, GSK-J4 efficiently induced cell death in PCa-derived OSCs.

**Conclusions:**

Our findings underscore the potential of the H19/cell adhesion molecules circuitry as a targeted approach in PCa treatment. Modulating this interaction has proven effective in inhibiting tumor growth and metastasis, presenting a logical foundation for targeted therapy.

**Supplementary Information:**

The online version contains supplementary material available at 10.1186/s12935-024-03231-6.

## Background

Prostate cancer (PCa) is widely spread worldwide, representing the most common cancer in men in western countries, with increasing incidence over the last decade [1, 2]. Current therapies reside on radical prostatectomy or radiotherapy with good results regarding the 5-year survival rate for localized prostate cancer [3]. However, approximately one-third of patients developed metastatic disease and relapsed after local therapy, with bone as the most common site of metastasis, followed by lung and liver [4–7]. Standard treatments for advanced prostate cancer rely on androgen-deprivation therapies (ADT), paralleled by association with some recently discovered novel hormonal agents [8]. Nevertheless, improving survival rates upon these new treatment modalities remains unsatisfactory [9], creating the need for local cytoreductive and metastasis-directed therapies [8, 10, 11] with economically-relevant consequences on the public health service.

Over the years, genetic and epigenetic alterations have been characterized in PCa, both associated with tumor initiation and disease progression [11–13]. Recently, the long non-coding RNAs (lncRNAs), > 200nt transcripts exerting essential biological functions mainly dysregulated in cancer, are highly involved in the epigenomic program by modifying histone and chromatin architecture as well as DNA and RNA [14]. In addition, mounting evidence in the literature supports the concept that either epigenetic deregulation or chronic inflammation might trigger the conversion of normal stem cells to cancer stem cells in some solid tumors, including PCa [15, 16]. In this view, lncRNA-H19 might play a significant role. LncRNA-H19 is a paternal-imprinted non-coding RNA, 2.3 kb in length, involved in embryonic development and stem cell growth [17, 18]. Indeed, aberrant lncRNA-H19 expression is associated with chronic inflammatory processes and several cancers [19, 20]. Overall, H19 affects cancer biology by various mechanisms, including epigenetic modifications, mainly by associating with the polycomb subunit enhancer of zeste homolog 2 (EZH2) [21–25]. Functionally, H19 can regulate tumor plasticity, acting as an oncogene or tumor suppressor depending on the cancer type and tumor microenvironment [24, 26, 27]. In prostate cancer cells, H19 over-expression reduces E-cadherin (*CDH1*) level and induces epithelial-to-mesenchymal transition. (EMT), exhibiting oncogenic properties [28]. On the contrary, H19 reduction, releasing expression of E-cadherin and β4 integrin (*ITGB4*), switches tumor dissemination toward an alternative mechanism, the cohesive phenotype, suggesting a tumor suppressor role for H19 in this condition [24]. In the cohesive metastatic phenotype, described by Harryman et al. [29], aggressive and metastatic PCa proceeds through a cluster of invasive cells expressing both integrins, for extracellular matrix remodeling during migration, as well as cadherins, for the cell-to-cell cohesion, supporting the so-called “collective migration”.

Epigenetic modulators have emerged as a compelling class of novel anti-cancer therapies capable of controlling crucial cellular functions such as proliferation, drug sensitivity, and resistance across various cancers, including PCa [30–32]. Epigenetic alterations involve changes in gene expression driven by chemical modifications to histone tails, DNA structure, or non-histone functional proteins. Predominant modifications encompass methylation, acetylation, and phosphorylation, facilitated by specific enzymes like Histone AcetylTransferases (HATs), Histone DeACetylases (HDACs), and Histone Methyltransferases (HMTs) [33]. For instance, the trimethylation of histone 3 lysine 27 (H3K27me3) transforms the chromatin structure from open to closed, subsequently diminishing gene transcription in the neighboring regions. The balance between histone lysine methyltransferase EZH2 and histone demethylases KDM6A/UTX and KDM6B/JMJD3 determines H3K27me3 levels. Inhibition of KDM6B/JMJD3 can decrease proliferation and induce apoptosis in cancer cells. In addition, KDM6B/JMJD3 knockdown may also affect the tumor progression program [34, 35].

Recently, the effects of the cell-permeable histone lysine demethylase inhibitor prodrug GSK-J4 [36] were reported as promising for developing novel therapies in castration-resistant prostate cancer (CRPC) [37–39]. Specifically, GSK-J4 inhibits both KDM6 subfamily members KDM6A/UTX and KDM6B/JMJD3 demethylases [36], being very efficient on the KDM6B/JMJD3-dependent cyclin D1 proliferation pathway [40]. Regarding the GSK-J4 targeting pathway, our previous study [24] elucidated a molecular mechanism in prostate cancer cells affecting specifically metastatic properties, named H19/cell adhesion circuitry. Characteristics of this circuitry are *(i)* H19-dependent repression of cell adhesion molecules transcription requires increasing H3K27me3 level; *(ii)* reduction of H19 under pro-tumoral stimuli, i.e. estrogen and hypoxia, increases both E-cadherin and β4 integrin, thus eliciting cohesive metastatic phenotype, and *(iii)* inhibition of demethylases by GSK-J4 reduces E-cadherin and β4 integrin expression and decreases in vitro metastatic potential.

The present study aims to investigate the impact of inhibiting the H19/cell adhesion circuitry using the histone demethylase inhibitor GSK-J4 on tumor growth and metastasis formation in vivo. We employed two mouse models: xenograft model with subcutaneous cell implant representing the straightforward model to study tumor growth and the experimental metastatic models with tail vein injection closely mimicking disease progression and metastatic process. We showed that treatment with GSK-J4 reduced tumor growth and metastasis formation in PCa tumor mouse models. In addition, GSK-J4 restored H19/cell adhesion molecules circuitry and induced cell death in freshly PCa-derived Organotypic Slice Cultures (OSCs), a three-dimensional experimental model recapitulating specific characteristics of the original donor patient.

Overall, our analysis with in vitro, in vivo, and ex-vivo experimental models demonstrated the pivotal role of the H19/cell adhesion molecules circuitry in driving the metastatic dissemination program in prostate cancer. This observation highlights its significance in PCa biology and supports the potential therapeutic application of the histone lysine demethylase inhibitor GSK-J4 in modulating tumor progression.

## Methods

### Antibody

AR (Millipore Cat# 06-680, RRID:AB_310214), ARv7 (Precision antibody Cat# AG10008, RRID:AB_2631057), Bcl-2 (R&D Systems Cat# AF810, RRID:AB_355621; Cat# MAB8272, RRID:AB_10890789; Dako clone 124 Cat# M0887, RRID:AB_2064429), β4 integrin (Abcam, Cambridge, UK, #ab133682, RRID:AB_2923284, and 450-11 A, RRID:AB_396065, as in [24], β-actin (Abcam Cat# ab8227, RRID:AB_2305186), E-cadherin (GeneTex Cat# GTX100443, RRID:AB_10729586 and Abcam, #ab231303, RRID:AB_2923285), Cytokeratin (Agilent Cat# GA053, RRID:AB_2892089 and Santa Cruz Biotechnology Cat# sc-81,714, RRID:AB_2191222), Goat-anti-Mouse Alexa Fluor 546 (Thermo Fisher Scientific Cat# A-11,003, RRID:AB_2534071), Goat anti-Mouse IgG HRP (Bio-Rad Cat# 170–6516, RRID:AB_11125547), Goat-anti-Rabbit IgG Alexa Fluor 546 (Thermo Fisher Scientific Cat# A-11,010, RRID:AB_2534077), Goat-anti-Rabbit IgG HRP (SeraCare KPL Cat# 5220 − 0336, RRID:AB_2857917), IgG (Bethyl Cat# P120-101, RRID:AB_479829), JMJD3 (Abcam Cat# ab38113, RRID:AB_943898), H3K27me3 (Active Motif Cat# 39,155, RRID:AB_2561020), HSP90 (Cell Signaling Technology Cat# 4877, RRID:AB_2233307), UTX (Abcam Cat# ab36938, RRID:AB_883400).

### Cell cultures, treatment, and transfection

PC-3M-luc2 were from Caliper Life,# 124,089 (RRID: CVCL_5J25) and provided by Prof. Carlo Leonetti (Istituto Nazione dei Tumori Regina Elena, Rome, Italy). 22Rv1-luc were generated from 22Rv1 (RRID: CVCL_1045) and kindly provided by Prof. Michael Henry (The University of Iowa, Iowa City, IA, USA) [41]. Western blot evaluated specific epithelial prostate markers, androgen receptors, full-length or ARv7 variant, and cytokeratin (Figure S1A). PC-3M-luc2 and 22Rv1-luc cells were grown in MEM (Corning, New York, USA, #15-010-CVR) and RPMI medium (1640 Corning, #10-040-CV), respectively, supplemented with 10% FBS (GIBCO, #10270106), 1% glutamine (Corning #25,005-CI), 1% penicillin and streptomycin (Corning #30002-CI). Medium for 22Rv1-luc cells was supplemented with 1% HEPES (Corning #25060-CI), 1% sodium pyruvate (Corning #25000-CIR), and 1% glucose. Cells were incubated at 37 °C with 5% CO_2_. GSK-J4 was prepared as described in [34]. Indirect (Hoechst) methods routinely screened all cell lines for mycoplasma contamination. The genetic identity of PC-3M-luc2 and 22Rv1-luc cell lines were authenticated by BMR Genomics (Padova, Italy) in October 2022. Cells were treated with GSK-J4 for the time and concentration indicated in the figure’s legend. Transient RNA interference for H19, KDM6A, and KDM6B was performed using TriFECTa Kit DsiRNA Duplex (Integrated DNA Technologies) according to the manufacturer’s instructions.

### H19 silencing and overexpression

PC-3M-luc2 and 22Rv1-luc cells were stable engineered using recombinant GFP-expressing lentiviral vectors for H19 silencing (siH19, Origene#TL318197V) compared to scramble vector (Vector, Origene#TR30021) or H19 overexpression (oeH19; Genecopoeia#LPP-CS-GS1189L-Lv201-01-050) compared to empty vector (EV, Genecopoeia#LP146-050). Lentiviral infection was performed using 2.5 × 10^6^ TU of lentivirus dissolved in 1 mL of medium supplemented with polybrene 8 µg/mL. 10^5^ cells were plated in a 6-well plate one day before infection. GFP expression was evaluated after five days post-transduction (Figure S1B).

### RNA extraction, cDNA preparation, real-time PCR, and droplet digital PCR

According to the manufacturer’s instructions, RNA from cells and tissues was extracted using Trizol (ThermoFisher). cDNA preparation and quantitative real-time PCR were performed as in [24] on QuantStudio 5 Real-Time PCR System (Applied Biosystems, Foster City, CA, USA) using SYBR Green quantification. The relative amount of each gene was measured as 2^−ΔCt^. β-Actin or P0 served as endogenous control. cDNA preparation, preamp PCR, and digital droplet PCR (ddPCR) were performed on QX200 droplet digital PCR system (Biorad) as described in [42]. Primers to H19, CDH1, ITGB4, P0, GAPDH, and β-actin as in [24], PSA as in [43]. Primers were as follows:

hKDM6A 5’-CAGTTAGCTTTGGTTGACTGTAATCC-3’ and 5’- AGTGGGCAATGTGAAATTGAATT-3’;

hKDM6B 5’-TGTGGAACTTGCTACACCTTGAG-3’ and 5’-GGTTCAGCAGCTCGCTTCAC-3’;

### Protein extraction and western blotting

Proteins were extracted and prepared using Trizol (ThermoFisher) as described in [24]. Western blots were performed using 15–20 µg of protein extract resolved by 4−12% gradient Invitrogen Precast gel (MES buffer) and revealed with an ECL Western Blot Detection Kit (Amersham Pharmacia Biotech, Buckinghamshire, England) using UVIDOC (Eppendorf S.r.l., Hamburg, Germany). Densitometry analysis was performed with ImageJ software (version 1.8.0).

### Immunofluorescence and confocal microscopy

Confocal analysis was performed as described in [44]. Briefly, cells were fixed in 4% paraformaldehyde and incubated with anti-E-cadherin antibody (Abcam, #ab231303) or β4 integrin (Abcam, #ab133682), diluted 1:250 in PBS 5% Goat Serum. Nuclei were counterstained with DAPI. Slides were analyzed with a confocal laser scanning system (Nikon Eclipse Ti2 confocal microscope) and Z stack images were processed by NIS Elements AR 5.30 software (Nikon Europe BV).

### Proliferation assay

Proliferation was assessed using the IncuCyte system S3 Kinetic Live Cell Imaging System (Essen BioScience, Ann Arbor, MI, USA). PC-3M-luc2 and 22Rv1-luc cells were seeded at 8000 and 15000 cells/well, respectively, in triplicate on a 24-well plate (Corning#3524), with IncuCyte readings taken at six h-cycles starting from day 0 (9 or 16 images per well). Pictures were taken every 2 or 4 h, and the IncuCyte algorithm was used for phase area confluence calculations.

### Trans-well invasion assays

Invasion assays were performed in triplicate as described in [24] with some modifications. Briefly, after 24 h in serum-free media, 1 × 10^5^ cells were seeded in top chambers of 24-well transwell plates (Corning #3422) in FBS-free media with membrane inserts with matrigel coated. Invading cells were stained with 0.1% crystal violet. Images were obtained by AXIO microscope with AxioCam Erc5s, Zeiss, under 20X or 10X magnification.

### Chromatin immunoprecipitation (ChIP)

ChIPs were performed as in [24], and analysis of DNA fragments was performed in duplicate by qPCR on QuantStudio 5 Real-Time PCR System (Applied Biosystems) using SYBR Master mix (Applied Biosystems, Foster City, CA, USA) with the evaluation of dissociation curves. Standard curves were generated by serially diluting the input (5-log dilutions in triplicate). The specific sequences isolated by the immune-complexes were normalized to the corresponding DNA input control, and data represented as relative enrichment. Immunoprecipitations were performed using specific antibodies to JMJD3 (Abcam, #ab38113), UTX (Abcam, #ab36938), and H3K27me3 (Active Motif #39,155, Carlsbad, CA, USA). IgG (Bethyl. #P120-101, Montgomery, TX, USA) was used as negative control. Primers for hCDH1 promoter were as in [24]. Primers for ITGB4 promoter were as follows:

hITGB4prom 5’- CTGGCCTGACACACACAGATCT-3’ and 5’-TTTGGGAACAATGTGGAAGGA-3’.

### Animal

NOD/SCID (RRID: IMSR_JAX:001303) and NSG (RRID: IMSR_JAX:005557) mice from Charles River Laboratories were housed 3–4 for cage in a room with controlled temperature, constant humidity, and a 12 h light/dark cycle with free access to food and water. The standard xenograft mouse model was generated using 5-6-week-old male NOD-SCID mice subcutaneously injected with 3 × 10^6^ cells/mouse with matrigel (1:1). Tumor growth was monitored by bioluminescence imaging (IVIS II Lumina, PerkinElmer Italy S.p.A., Milan, Italy). Data were acquired and analyzed using the living image software version 4.7.4 (Caliper Life Sciences). Tumor growth was measured with digital calipers, and tumor volumes were calculated from the formula V = (W^2^ x L)/2, W = width, L = length. In addition, a metastatic mouse model was generated by injecting 1 × 10^6^ cells into the lateral tail vein of NSG mice (5–6 weeks old). Mice were randomly divided into two groups and treated with GSK-J4- (50 mg/Kg diluted 10:90 DMSO:20% captisol as in [45] and administrated via IP 5 days/week) or vehicle-treated (DMSO) starting from day 0. At the end of the experiments, subcutaneous tumors were collected for further analysis, and metastases on different organs were visualized and quantified by ex vivo bioluminescence using IVIS Lumina.

### Histological analysis

For histological and immunohistochemical analyses, tissues were fixed in 10% formalin (Thermo Fisher Scientific). Unstained tissue Sect. (4 μm-thick) were cut from formalin-fixed, paraffin-embedded blocks and mounted on a positively charged glass slide. IHC was performed using the Leica Bond (LBO, Milan, Italy) immunostainer or a Ventana Benchmark XT automated immunostainer (Ventana Medical Systems, Tucson, AZ, USA) as follows: cytocheratin AE1/AE3, Agilent DAKO, #GA053, ER pH6 for 20 min. Hematoxylin was used for nuclear counterstaining. Whole slide imaging (WSI) was performed using NanoZoomer 2.ORS (Hamamatsu photonics, Hamamatsu, Japan) using 20X magnification (0.46 microns/pixel).

### Organotypic slice cultures (OSCs)

PCa patients (*n* = 25) were enrolled at the Urology of Università Cattolica (Rome, Italy) to perform prostatectomy with the following inclusion criteria: (i) clinically localized PCa at diagnosis and (ii) absence of hormone treatment/radiotherapy before surgery. Of note, all OSCs were checked by the pathologist on the original histopathological slide for morphology, tissue architecture, and amount of tumor (≥ 75%). OSCs were generated as previously described in [24, 43, 46]. Briefly, medium was replaced daily and collected for cell death assay (see paragraph below). Slices were treated with GSK-J4 (5µM) for 72 h, and RNA/protein was extracted and analyzed as previously described [24].

### Cell death assay

Apoptosis was determined with Cell Death Detection ELISA PLUS kit (Roche, Basel, Switzerland) using 5–20 µl extracellular-medium according to manufacturers’ instructions. Absorbance at 405 and 490 nm was assessed at VICTOR X4 (Perkin Elmer).

### Statistical analysis

Data were expressed as mean ± SEM or as fold change as indicated in figure legends. Difference among ≥ 3 groups were analyzed with a Kruskal-Wallis test, and post hoc comparison was done using the Mann-Whitney U test (α = 0.05). Difference among 2 groups were analyzed with Mann-Whitney U test. Chi-square test were used to compare the proportion of the number of mets/mouse between groups as indicated in figure legend. Statistical analysis was performed using GraphPad Prism 8.0.2 statistical software. *P*-values of < 0.05 were considered significant.

## Results

### H19/cell adhesion molecules circuitry governs in vitro metastatic potential in PC-3 and 22Rv1 cells

The experimental evidence that H19/cell adhesion molecules circuitry is involved in the acquisition of aggressive phenotype, i.e. Cohesive metastatic phenotype, potentially identifies this circuitry as a novel molecular target therapy. As first step to evaluate the in vivo efficacy of GSK-J4, the H19/cell adhesion molecules circuitry was analyzed in PCa-derived cells to develop reliable mouse tumor models: specifically, the luciferase positive PC-3 and the 22Rv1 prostate cancer cell lines, derived from a human metastatic lesion and a xenograft serially propagated in mice after castration-induced regression and relapse of the parental, androgen-dependent CWR22 xenograft, respectively. Of note, these cell lines represent two models of advanced prostate cancer characterized by the lack of androgen receptor (AR-null) or by the co-expression of the AR variant (ARv7) and AR full length, PC-3 and 22Rv1, respectively.

PC-3luc cells have transiently interfered with siH19 or negative control (NC1), obtaining a good targeting of H19 level (about 40%, Figure S2A, left panel). Consequently, cell adhesion molecules E-cadherin and β4 integrin were induced at mRNA and protein levels (ranging from 2- to 4-fold, Figure S2A and B, respectively). Of note, H19 silencing increased invasion capability about 2-fold (Figure S2C). Interestingly, the H19/cell adhesion molecules circuitry was unaffected by the ARv7 variant as observed in Figure S2, right panels: H19 silencing in 22Rv1-luc cells induced indeed expression of E-cadherin and β4 integrin at mRNA and protein level (ranging from 2 to 4-fold, Figure S2A and B, respectively) as well as the number of invading cells (Figure S2C).

**Fig. 1:**
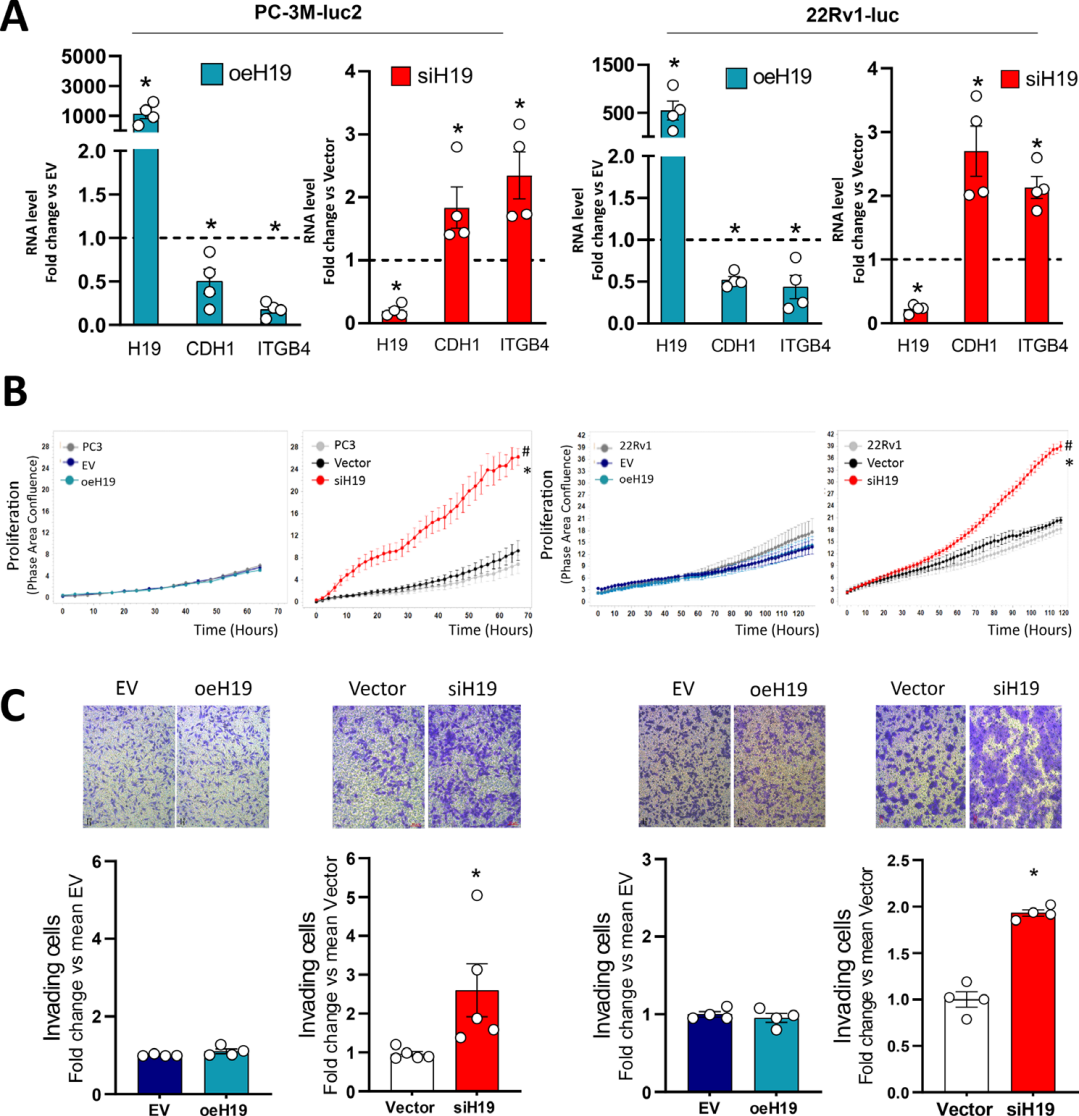
Evaluation of in vitro metastatic potential in PC-3 and 22Rv1 cells after H19 silencing or overexpression. PC-3M-luc2 (left) and 22Rv1-luc (right) cells were subjected to lentiviral transduction to obtain stable H19 silencing (siH19) compared to scramble vector (Vector) and H19 overexpression (oeH19) compared to empty vector (EV). (**A**) H19, E-cadherin (CDH1), and β4 integrin (ITGB4) RNAs were assessed by qPCR. Data, plotted as fold change vs. Vector or EV (dashed line), represent the mean ± SEM of 4 independent experiments (white dots). *P < 0.05 vs. Vector or EV. (**B**) Proliferation assay at different time points. Cells were monitored using the IncuCyte live cell analysis system. Cell confluence was calculated from raw data images. Data represent the mean ± SEM of 3 independent experiments performed in triplicate. *P < 0.05 vs. Vector; ^#^ *P* < 0.05 vs. PC-3 or 22Rv1. (**C**) Trans well Cell invasion assay after 16 h. Upper panel: representative phase-contrast microscopic images of invading cells under 20X magnification (bright field). Scale bar: 20 μm. Lower panel: number of invading cells. Data plotted as fold change vs. mean vector represent the mean ± SEM of 4 independent experiments (white dots). *P < 0.05

Next, stable H19 silencing or overexpression cells were obtained by infection with recombinant lentiviral vector carrying H19 shRNA interfering (siH19) compared to scramble vector (Vector), H19 full length (oeH19) compared to empty vector (EV) in both PC3 and 22Rv1 luciferase positive cells (PC-3M-luc2 and 22Rv1-luc, Fig. 1). Levels of H19 and, in turns, of E-cadherin, and β4 integrin at RNA and protein levels by Western Blot and confocal microscopy are shown in Fig. 1A and Fig S3, respectively. As expected, overexpression of H19 reduced E-cadherin and β4 integrin levels in oeH19 cells (Fig. 1A and Figure S3A and C), while silencing of H19 induced E-cadherin and β4 integrin levels in both PC-3 and 22Rv1 (Fig. 1A and Figure S3B and D). In addition, siH19 cells presented a higher proliferation rate than vector or not transfected PC-3 or 22Rv1 cells (Fig. 1B), while oeH19 cells showed similar proliferation rate compared to control EV and not transfected PC-3 or 22Rv1 cells (Fig. 1B). H19 silencing increased metastatic potential in PC-3 and 22Rv1 cells as assessed by invasion assay (about 2-fold, Fig. 1C).

These results demonstrated that the H19/cell adhesion molecules circuitry is active in both cell lines regardless androgen pathway and governs in vitro metastatic potential: specifically, H19 silencing increases in vitro metastatic potential.

### GSK-J4 treatment reduced cell adhesion molecules expression and in vitro metastatic potential in H19-silenced PCa cells

**Fig. 2:**
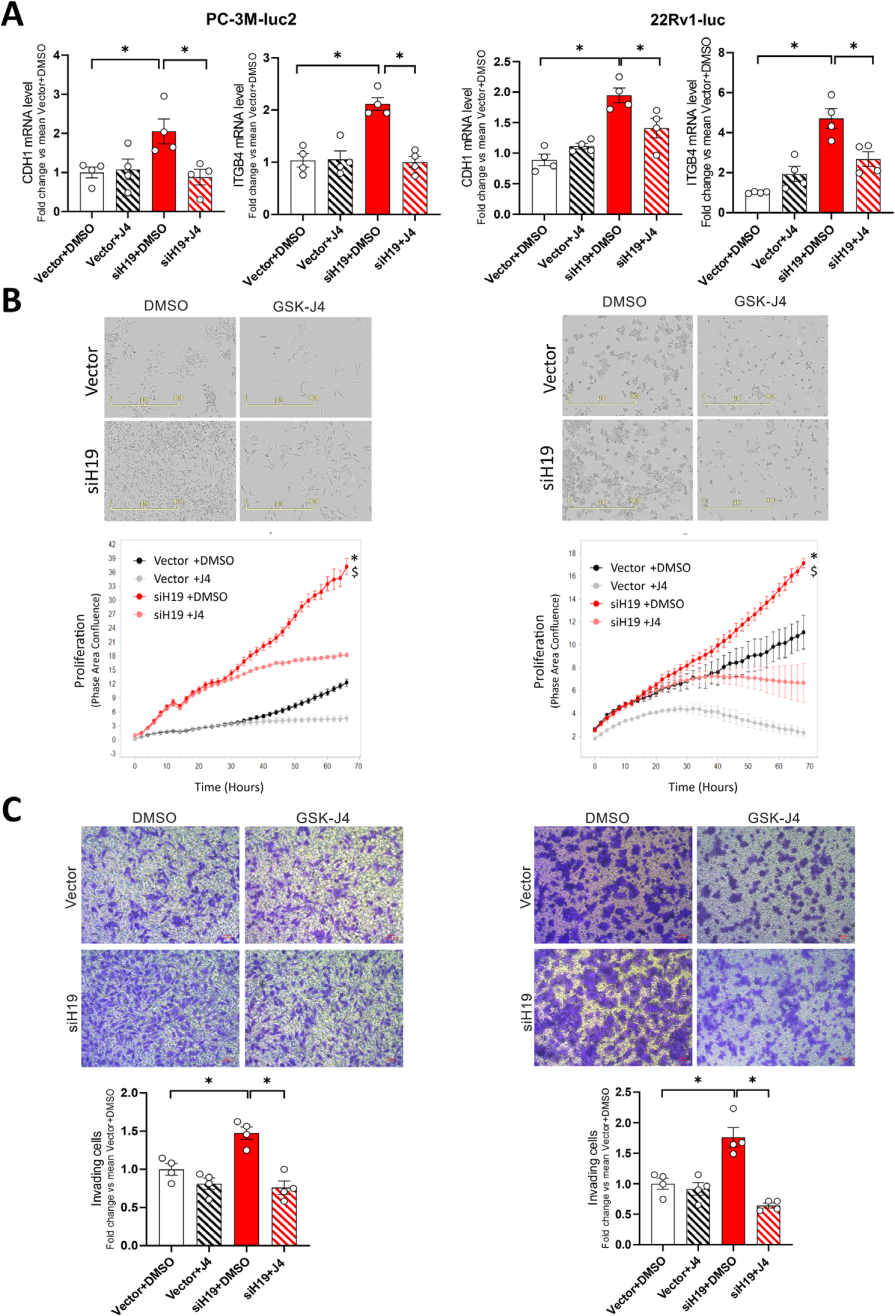
Efficacy of GSK-J4 on cell adhesion molecules expression, proliferation, and invasiveness in siH19 cells. H19-silenced (siH19) or control (Vector) PC-3M-luc2 (left) and 22Rv1-luc (right) cells were treated for 72 h with GSK-J4 demethylases inhibitor (1µM) or DMSO as control. (**A**) CDH1 and ITGB4 mRNA levels were assessed by qPCR. Data plotted as fold change vs. mean Vector + DMSO represent the mean ± SEM of 4 independent experiments (white dots). (**B**) Cell proliferation was monitored using the IncuCyte live cell analysis system. Upper: raw data pictures of cell confluence exported from the IncuCyte system after 48 h incubation; scale bar is indicated; Lower: Cell confluence was calculated from raw data images; data shown is a representative experiment of 4 biological replicates, each time point represent the mean of 4 samples. *P < 0.05 vs. Vector + DMSO; ^$^ *P* < 0.05 vs. siH19 + J4. (**C**) Cell invasion by Trans well assay. Upper: representative phase contrast microscopic images of invading cells under 20X magnification (bright field). Scale bar: 20 μm. Lower: number of invading cells. Data plotted as fold change vs. mean Vector + DMSO represent the mean ± SEM of 4 independent experiments (white dots). *P < 0.05

To investigate whether the modulation of H19/cell adhesion molecules circuitry might be used as a novel targeted therapy for aggressive prostate cancer, epigenetic interference of JMJD3/UTX histone demethylases was evaluated on in vitro cell growth and metastasis potential before and after H19 silencing. Treatment with the histone lysine demethylases inhibitor GSK-J4 significantly reduced both E-cadherin and β4 integrin levels in H19-silenced PC-3 and 22Rv1 cells (siH19) compared to control (Vector) at both mRNA and protein levels restoring the basal level of cell adhesion molecules (Fig. 2A and S4, respectively). Of note, GSK-J4 treatment significantly reduced cell proliferation and invasion capability in siH19 cells compared to control (Fig. 2B and C) while induced apoptosis, as assessed by the decrease of Bcl2 level and induction of cell death (Figure S5A, B and C, respectively). Similar results were also observed after transient H19 silencing in PC-3M-luc2 cells (Figure S6).

To investigate whether the effect of GSK-J4 treatment was mediated by KDM6A/UTX or KDM6B/JMJD3 demethylase, transient silencing was performed in siH19 compared to Vector control in PC-3M-luc2 cells (Figure S7). In addition, KDM6A and 6B targeting were evaluated by mRNA and protein level; both were efficiently reduced at 70–80% at 72 h post-transfection (Figure S7A and B, respectively).

**Fig. 3:**
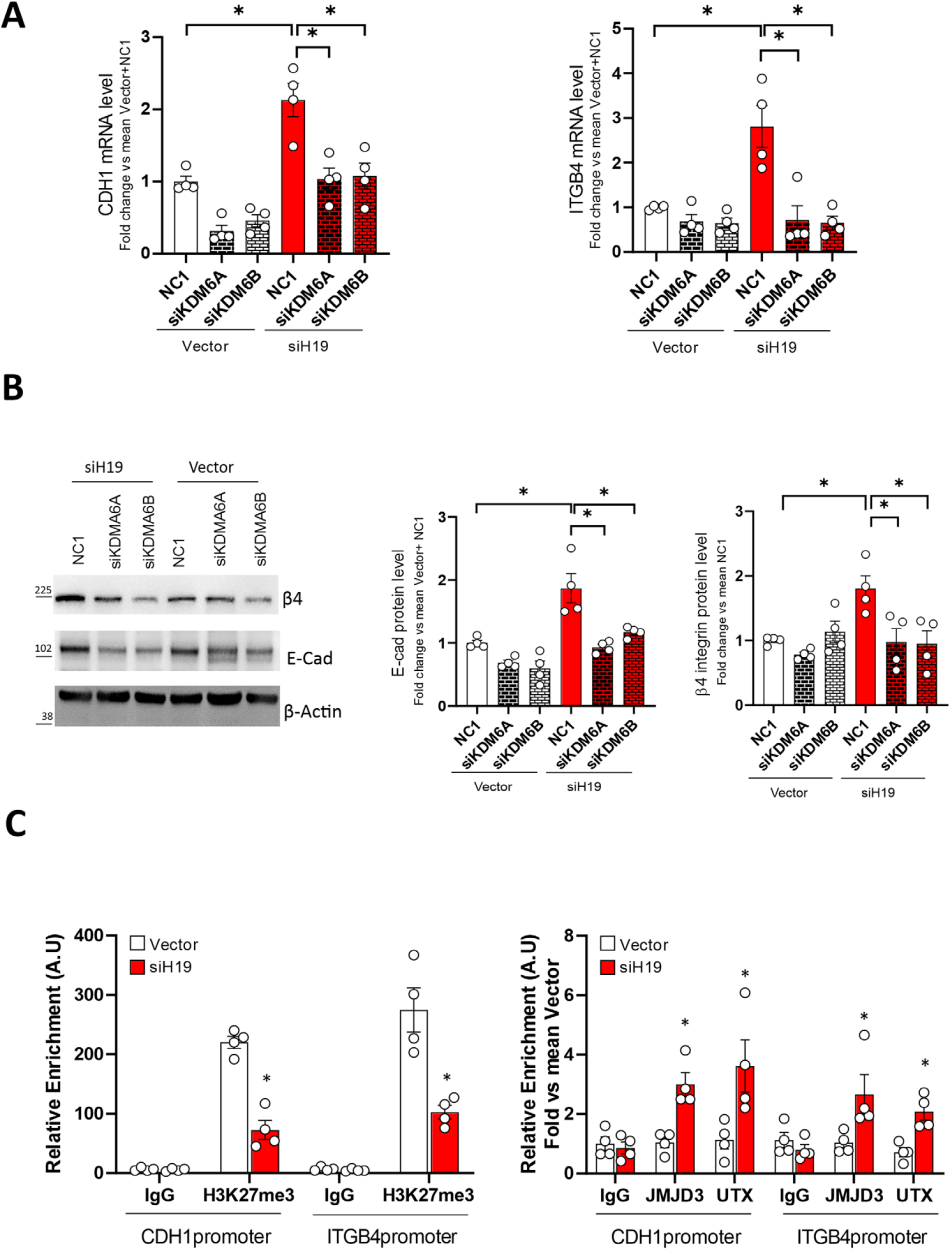
Effects of KDM6A and KDM6B silencing on cell adhesion molecules expression in siH19 cells. SiH19 and Vector cells were transfected with siRNA specific for KDM6A and KDM6B or scramble (NC1), and analysis was performed after 72 h. (**A**) CDH1 and ITGB4 mRNA levels were quantified by qPCR. Data are represented as mean ± SEM of fold change vs. mean Vector/NC1 cells of 4 independent experiments (white dots). *P < 0.05. (**B**) Representative E-Cad and β4-integrin western blot (left panels) and densitometry analysis (right panels) after KDM6A and KDM6B interfering. β-actin served as control. Molecular weight marker is indicated. Data are represented as mean ± SEM of fold change vs. mean Vector/NC1 cells of 4 independent experiments (white dots). *P < 0.05. (**C**) Enrichment of H3K27me3 (left) and recruitment of KDM6A/UTX and KDM6B/JMJD3 (right) on the promoter region of CDH1 and ITGB4 by ChIPs. IgG served as the negative control. Values represent the mean ± SEM of 4 independent experiments. Data are plotted as Relative enrichment relative to Input in Arbitrary Unit (A.U.) or fold vs. mean Vector. *P < 0.05 vs. Vector

Here, we evaluated E-cadherin and β4 integrin at mRNA and protein levels. Figure 3A and B show that KDM6A and KDM6B silencing restored basal levels of both cell adhesion molecules in siH19 cells compared to vector control. In addition, both CDH1 and ITGB4 promoters were analyzed by chromatin immunoprecipitation (ChIP) in siH19 cells compared to Vector control cells (Fig. 3C). Chromatins were immunoprecipitated by antibodies to H3K27me3, KDM6A/UTX, and KDM6B/JMJD3 demethylases, and IgG as negative control and proximal promoters were amplified. In line with the above results, H3K27me3 level on the promoter of both CDH1 and ITGB4 were reduced (about 50%) while recruitment of KDM6A/UTX and KDM6B/JMJD3 increased in siH19 cells compared to control (range 2- to 4-fold).

These results suggested that both KDM6A/UTX and KDM6B/JMJD3 demethylases regulate E-cadherin and β4 integrin in PCa cells and that the epigenetic interfering of the H19/cell adhesion molecules circuitry with the demethylase inhibitor GSK-J4 possess the potential of a novel targeted therapy for aggressive PCa, regardless androgen responsiveness/androgen receptor status.

### GSK-J4 treatment reduced tumor growth in a subcutaneous murine xenograft model

**Fig. 4:**
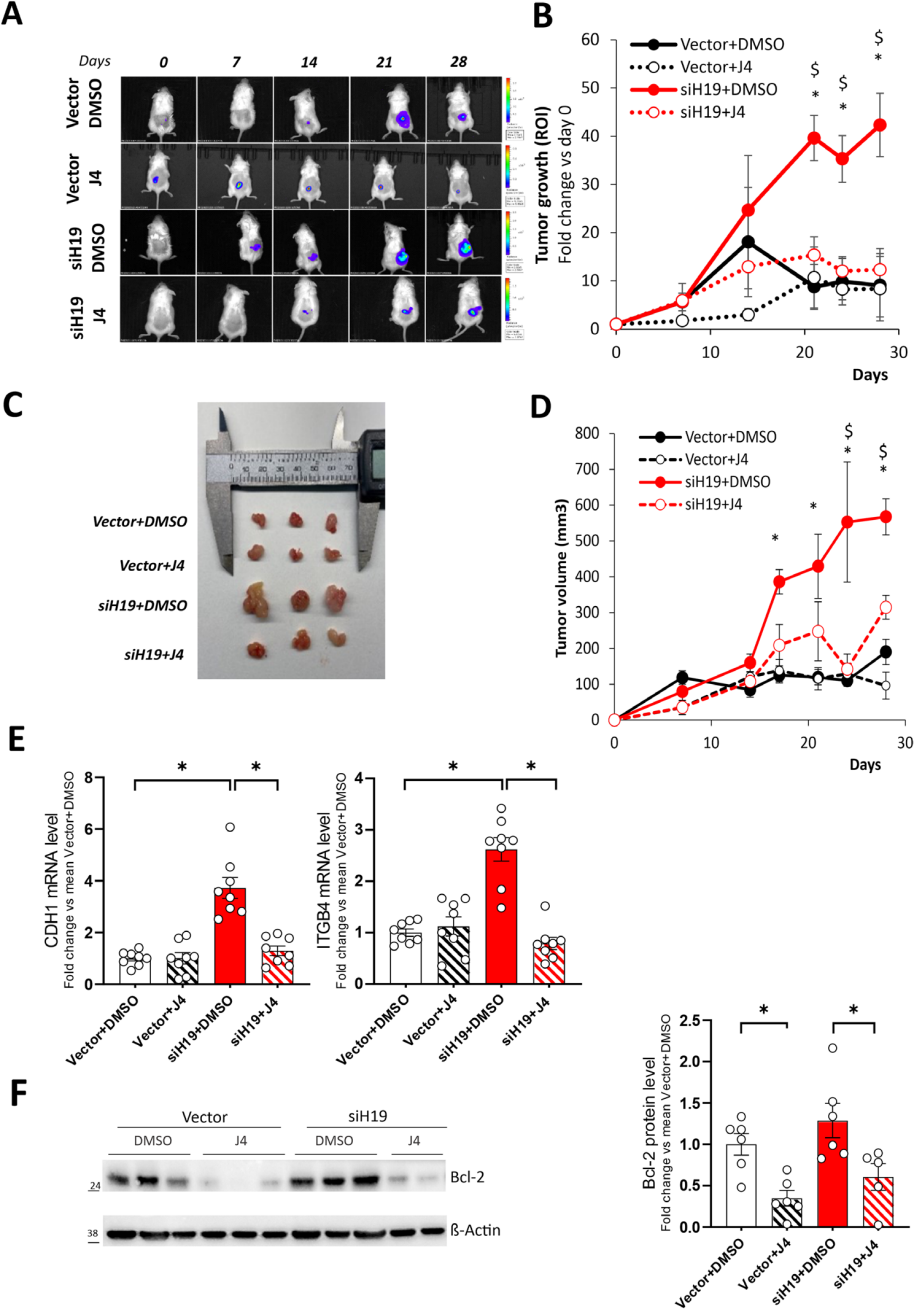
Effect of GSK-J4 treatment on a subcutaneous xenograft mouse model of H19 silenced PC-3M-luc2 cells. (**A**) Sequential in vivo imaging of tumor growth post subcutaneous injection of siH19 and control Vector cells in NOD/SCID mice treated with GSK-J4 (J4) or vehicle (DMSO). Panels depict a representative mouse from each group. (**B**) Tumor growth was measured as photons/sec in the region of interest (ROI). Data plotted as fold change vs. day 0 represent mean +/- SEM of 8 mice/group. *P < 0.05 vs. Vector + DMSO; ^$^ <0.05 vs. siH19 + J4. (**C**) Ex vivo photos of representative solid tumors on the day of the explant. (**D**) Tumor volume was evaluated by caliper measurements at the different time points and calculated as follows: V (mm^3^) = (W^2^ × L)/2. Data represent mean +/- SEM of 8 mice/group. *P < 0.05 vs. Vector + DMSO;^$^ <0.05 vs. siH19 + J4 (**E**) CDH1 and ITGB4 mRNA analyzed by qPCR in tumor samples. Data, represented as fold change vs. mean Vector + DMSO, represent the mean +/-SEM of 8 mice/group (white dots). *P < 0.05. (**F**) Representative western blot and relative densitometric analysis for bcl-2 in tumor samples. β-actin was used as a loading control. Molecular weight marker is indicated. Data plotted as fold change vs. mean Vector + DMSO represent mean ± SEM of 5-6 mice/group (white dots). *P < 0.05

To investigate in vivo the effect of GSK-J4 demethylases inhibitor, siH19 PC-3luc and control Vector cells were inoculated subcutaneously into the right flank of NOD/SCID mice, mice divided into two groups, GSK-J4- (J4) and vehicle-treated (DMSO), and tumor growth monitored by in vivo bioluminescence imaging and calipers. Tumor growth produced by siH19 cells implantation was significantly enhanced compared to control Vector or oeH19 (Fig. 4A and Figure S8A and B, respectively), and GSK-J4 treatment restored basal growth as assessed by bioluminescent intensity (Fig. 4A and B) and tumor volume (Fig. 4C and D). Of interest, in vivo GSK-J4 treatment reduced the expression of CDH1 and ITGB4 mRNA and bcl-2 protein levels in siH19-subcutaneous tumors (Fig. 4E and F). Notably, H19 silencing in 22Rv1-luc cells also enhances tumor growth in the subcutaneous murine xenograft model (Figure S8C and D).

These results suggested that in vivo GSK-J4 treatment targeting H19/cell adhesion molecules circuitry might affect the growth of prostate cancer.

### GSK-J4 treatment reduced metastasis dissemination in an experimental metastasis mouse model

**Fig. 5:**
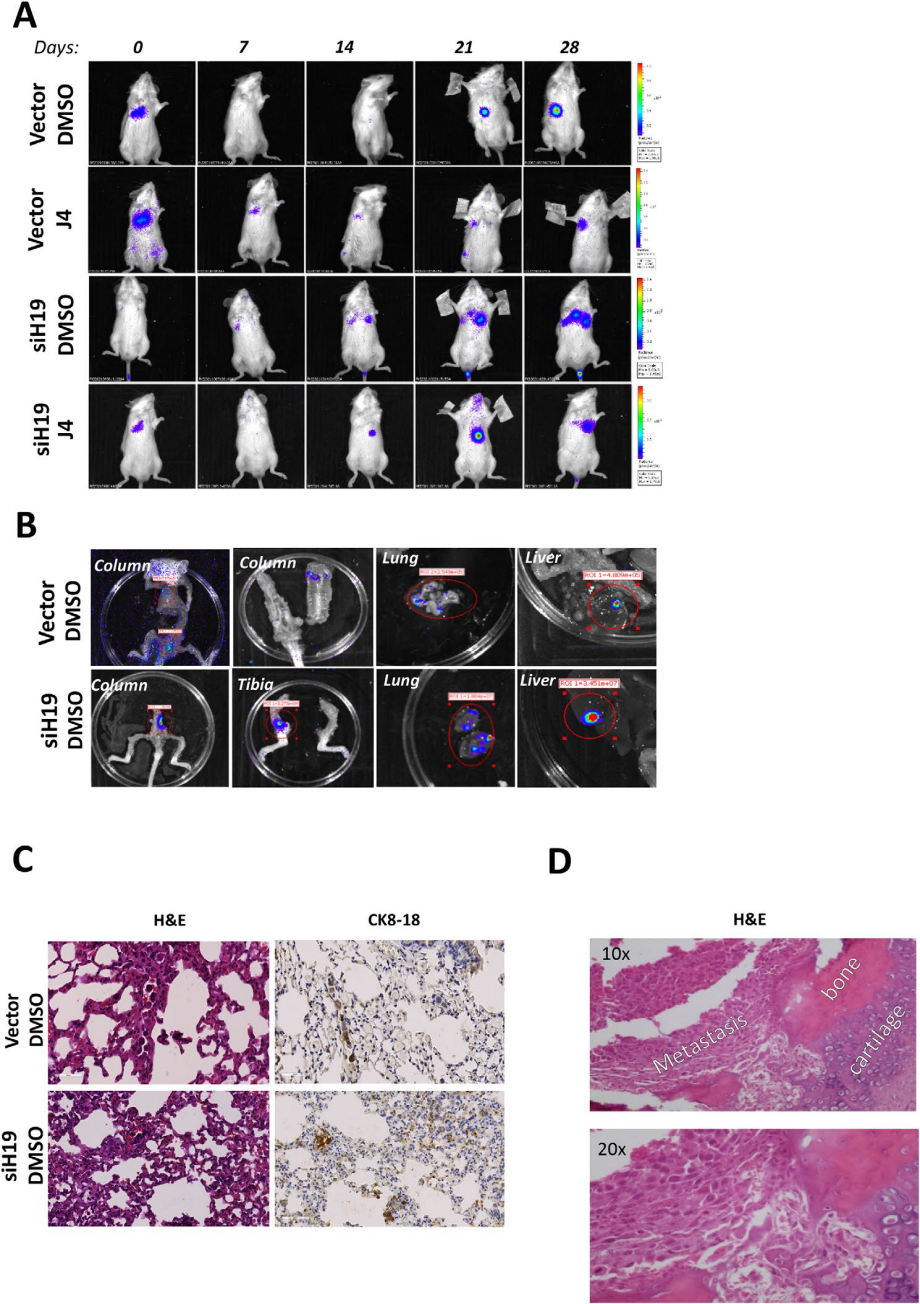
Metastatic dissemination of PC-3M-luc2 cells after H19 silencing. (**A**) Sequential in vivo imaging of metastatic tumor dissemination over time post intravenous injection of siH19 and control Vector cells in NGS mice in the presence or absence of GSK-J4 (50 mg/kg) or DMSO as control (*n* = 12 mice/group). (**B**) Representative ex vivo bioluminescence images in different dissemination sites (column, tibiae, lung, and liver). (**C**) Representative sections H&E-stained (left) and IHC for human cytokeratin CK8-18 (right) sections of the lung with Vector and siH19 cells. Scale bar: 50 μm. (**D**) Representative H&E-stained sections of bone metastasis under 10X and 20X magnification. Metastatic cells, bone, and cartilage, are indicated.

Bone, lung, and liver are the most common metastatic sites in advanced human prostate cancer [6, 7]. To address whether GSK-J4 treatment affects in vivo metastatic dissemination, siH19 PC-3 and control cells were injected into the lateral tail vein of NSG mice, mice divided into two groups, GSK-J4- (J4) and vehicle-treated (DMSO), and establishment of a colony in tissues monitored by in vivo bioluminescence imaging as shown in Fig. 5A, both siH19 and Vector cells metastasized to the lung. In addition, bone and liver metastasis were also observed at the end of the experiment (28 days after injection) when whole organs were removed and scanned ex vivo using an IVIS imaging system (Fig. 5B).

Of note, Immunohistochemistry (IHC) on lungs revealed the presence of several micrometastases with peculiar patterns (Fig. 5C and Figure S9A). Specifically, in siH19-injected mice, we observed clusters of human cells embedded in mouse lung tissues (Fig. 5C) as well as in lung blood vessels (Figure S9A) in agreement with the previously described cohesive metastatic phenotype or collective migration [24, 29]. Control Vector cells appeared as individual cells or as smaller clusters. Staining with specific antibody anti-human pan-cytokeratin confirmed the distribution of metastatic cells embedded in murine lung tissues (Fig. 5C). In parallel, IHC on column and tibia metastasis showed a cluster of human cells in murine bones and cells invading bone and cartilage matrix (Fig. 5D and Figure S9B and C).

**Fig. 6:**
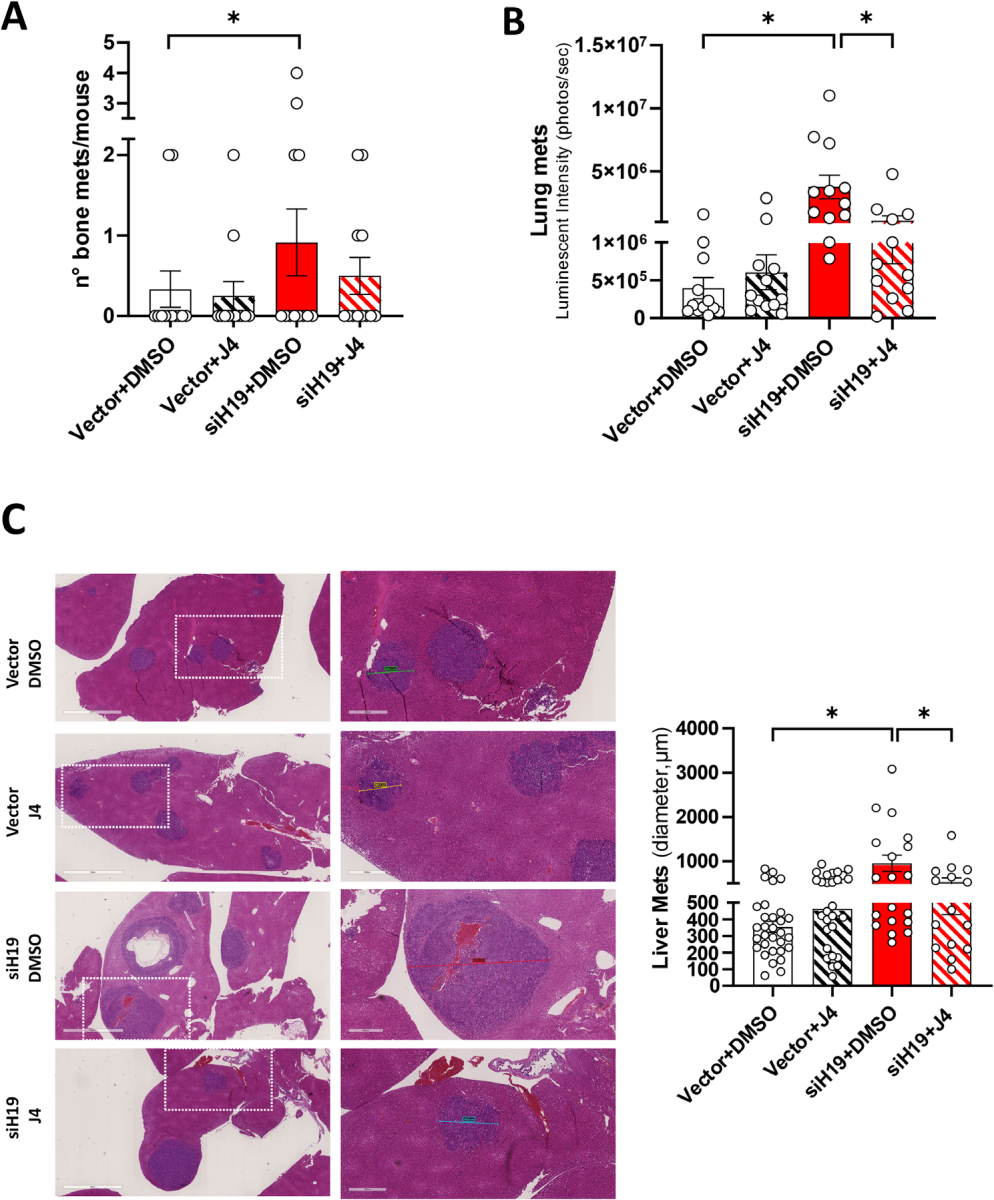
Effects of GSK-J4 on a metastatic mouse model. Tumor metastatic dissemination post intravenous injection of siH19-PC-3 and Vector cells in presence or absence of GSK-J4 as in the legend to Fig. 5. (**A**) Number of bone metastasis (mets)/mouse. Data represent the mean +/- SEM of 12 mice/group (white dots). Statistical significance was determined by the Chi-square test, 2-tail, on the proportion of the number of mets/mouse. *P < 0.05. (**B**) Lung metastasis measured as photons/sec in Region Of Interest (ROI) on the day of explant. Data represent mean +/- SEM of 12 mice/group (white dots). (**C**) Representative H&E-stained sections of the liver. Dashed lines indicated the zoom area in the right panels. Color lines indicate metastasis diameter. Scale bars:2 mm and 500 μm. Right: Tumor growth measured in the liver as diameter (µm) on H&E staining. Data represent mean +/- SEM of 12 mice/group (2 or 3 metastasis/mouse, white dots). *P < 0.05

Of interest, siH19 cells could produce a higher bone metastasis formation, both the number of bone metastasis/mouse and total bone metastasis (Fig. 6A), as well as lung metastasis (Fig. 6B), compared to Vector control cells. In addition, siH19 cells produce liver metastasis with increased diameter (Fig. 6C). Importantly, treatment with GSK-J4 reduced metastasis formation produced by siH19 cells in bone, lung, and liver (Fig. 6B and C).

These results indicated that GSK-J4 treatment might reduce in vivo metastatic properties and may represent a potential novel target therapy for aggressive prostate cancer.

### GSK-J4 treatment affects H19/cell adhesion molecules expression in Organotypic slice cultures (OSCs)

**Fig. 7:**
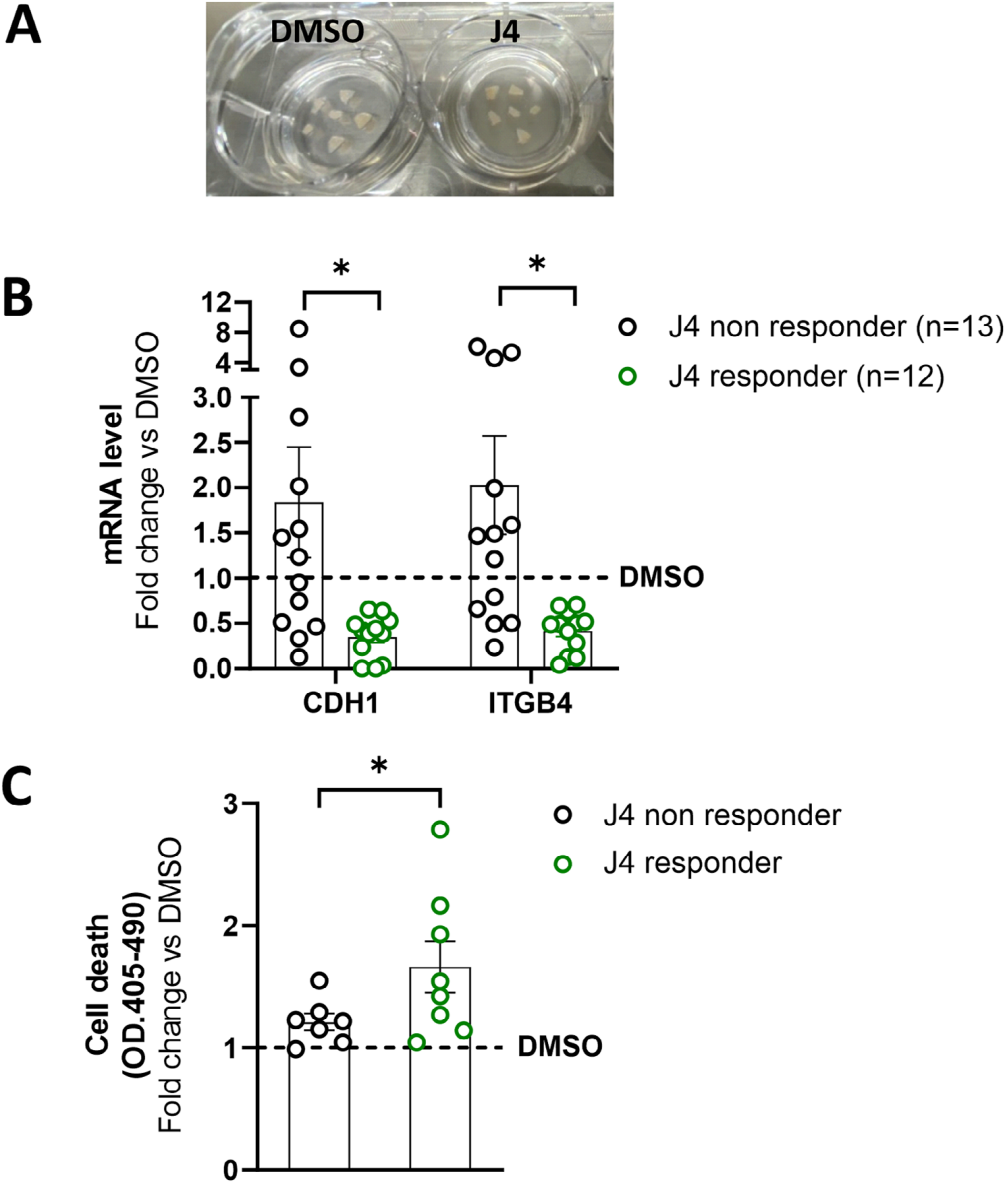
Effects of GSK-J4 on Organotypic Slices Cultures. (**A**) Representative images of OSC after 72 h treatment with GSK-J4 (J4, 5µM) or DMSO as control. (**B**) Quantification of CDH1 and ITGB4 transcripts by qRT-PCR (total OSC *n* = 25). OSCs were divided into J4-responder or J4-non responder according to a 25% reduction upon treatment. Data plotted as fold change vs. DMSO (dashed line) represent mean +/-SEM, and white dots indicate a single OSC. *P < 0.05 vs. DMSO. (**C**) Apoptosis induction upon GSK-J4 treatment evaluated using Cell Death Detection ELISA Kit as described in Methods. Data are expressed as fold change vs. DMSO (*n* = 15). *P* < 0.05 vs. DMSO

To assess the effect of GSK-J4 treatment on human prostate cancer tissues, human PCa-derived Organotypic Slice Cultures (OSCs) were selected as a reliable ex vivo model. OSCs, obtained from fresh surgical explants of organ-confined prostate tumors, represent a relevant three-dimensional experimental model recapitulating specific characteristics of original tissue [24, 43, 46, 47] (Fig. 7A). OSCs were obtained from a cohort of 25 PCa patients (Table 1) with localized disease undergoing surgery from January 2020 to June 2022 at the Urology of Università Cattolica (Rome, Italy).

**Table 1:** Clinical and pathologic features of patients and tumors used for the analysis of OSCs

PCa Patients	Age	PSA (ng/ml)	Pathological Gleason Score	Pathological Stage
osc#52	69	6,2	7 (3 + 4)	pT3a pNx pMx
osc#53	63	13.8	7 (3 + 4)	pT3a pN0 pMx
osc#54	66	2,4	7 (3 + 4)	pT3a pNx pMx
osc#56	64	20	7 (3 + 4)	pT2c pNx pM0
osc#57	77	15.2	9 (4 + 5)	pT3b pN0 pMx
osc#58	73	6.7	7 (3 + 4)	pT3a pN1 pMx
osc#59	70	8.2	7 (3 + 4)	pT2 pN0 pMx
osc#63	67	8	7 (3 + 4)	pT2c pNx pMx
osc#64	62	13	7 (3 + 4)	pT3a pN0 pMx
osc#65	75	7.6	7 (3 + 4)	pT2c pN0 pMx
osc#66	71	9.9	7 (4 + 3)	pT3b pN1 pMx
osc#67	69	9	7 (3 + 4)	pT2c pN0 pMx
osc#68	69	5	7 (4 + 3)	pT3a pN0 pMx
osc#69	68	5	7 (3 + 4)	pT3a pN0 pMx
osc#70	64	6,7	7 (3 + 4)	pT2c pNx pMx
osc#71	71	4,8	7 (3 + 4)	pT2c pN0 pMx
osc#72	59	5	7 (3 + 4)	pT2c pNx pMx
osc#73	57	5.4	7 (3 + 4)	pT2c pNx pMx
osc#74	69	3.9	7 (3 + 4)	pT2c pNx pMx
osc#75	79	17	7 (4 + 3)	pT3a pNx pMx
osc#76	75	7	7 (4 + 3)	pT2c pN0 pMx
osc#77	49	5	7 (3 + 4)	pT2c pNx pMx
osc#78	77	11.9	7 (4 + 3)	pT3a ,pNx,pMx
osc#79	74	5.1	7 (4 + 3)	pT3b,pNx,pMx

Cell adhesion molecules expression was assessed in OSCs treated for 72 h with GSK-J4 or vehicle (DMSO) at mRNA level (Fig. 7B and Figure S10A). GSK-J4 treatment significantly reduced CDH1 or ITGB4 mRNAs in 16/25 and 14/25 OSCs, respectively (Figure S10). OSCs were divided into two groups: J4-responder (with at least 25% significant reduction in both CDH1 and ITGB4 mRNA level, *n* = 12) and J4-non responder (*n* = 13, Fig. 7B and Figure S10). Of note, GSK-J4 induced cell death in J4-responder compared to J4 non-responder OSCs (up to 1.7-fold increase) as assessed by detecting histone-associated DNA fragments released in extracellular-medium after 72 h treatment (Fig. 7C).

Overall, these results show, at least as proof of principle, that GSK-J4 affects H19/cell adhesion molecules circuitry also in the ex vivo PCa samples and, importantly, are in agreement with data obtained in PCa cell lines and mouse tumor models.

## Discussion

Despite significant efforts, metastatic PCa remains largely incurable with a relevant impact on the public health service. In this study, we aimed to elucidate the crucial role of H19/cell adhesion molecules circuitry in the metastasis dissemination on in vivo and ex vivo PCa experimental models. From a molecular point of view, H19 acts as a transcriptional repressor of cell adhesion molecules by increasing the H3K27me3 level at the corresponding promoter regions. Upon H19 reduction, transcription of CDH1 and ITGB4 is released, increasing metastatic potential, and metastasis dissemination through a mechanism also known as “cohesive metastatic phenotype” described for prostate cancer [24, 29] as well as for other cancer types like colorectal cancer and squamous cell carcinomas as recently reported [48, 49]. A cluster of tumoral cells, linked together by E-cadherin and capable of motility through β4 integrin expression and function, is present in lung parenchyma and lung vessels after injecting siH19 cells in the tail vein of NSG mice (Fig. 5C and S9A). Interestingly, this phenomenon is linked, at least in prostate cancer, to pro-tumoral stimuli as estrogens and hypoxia that specifically reduced H19 gene expression in PCa cells [24].

**Fig. 8:**
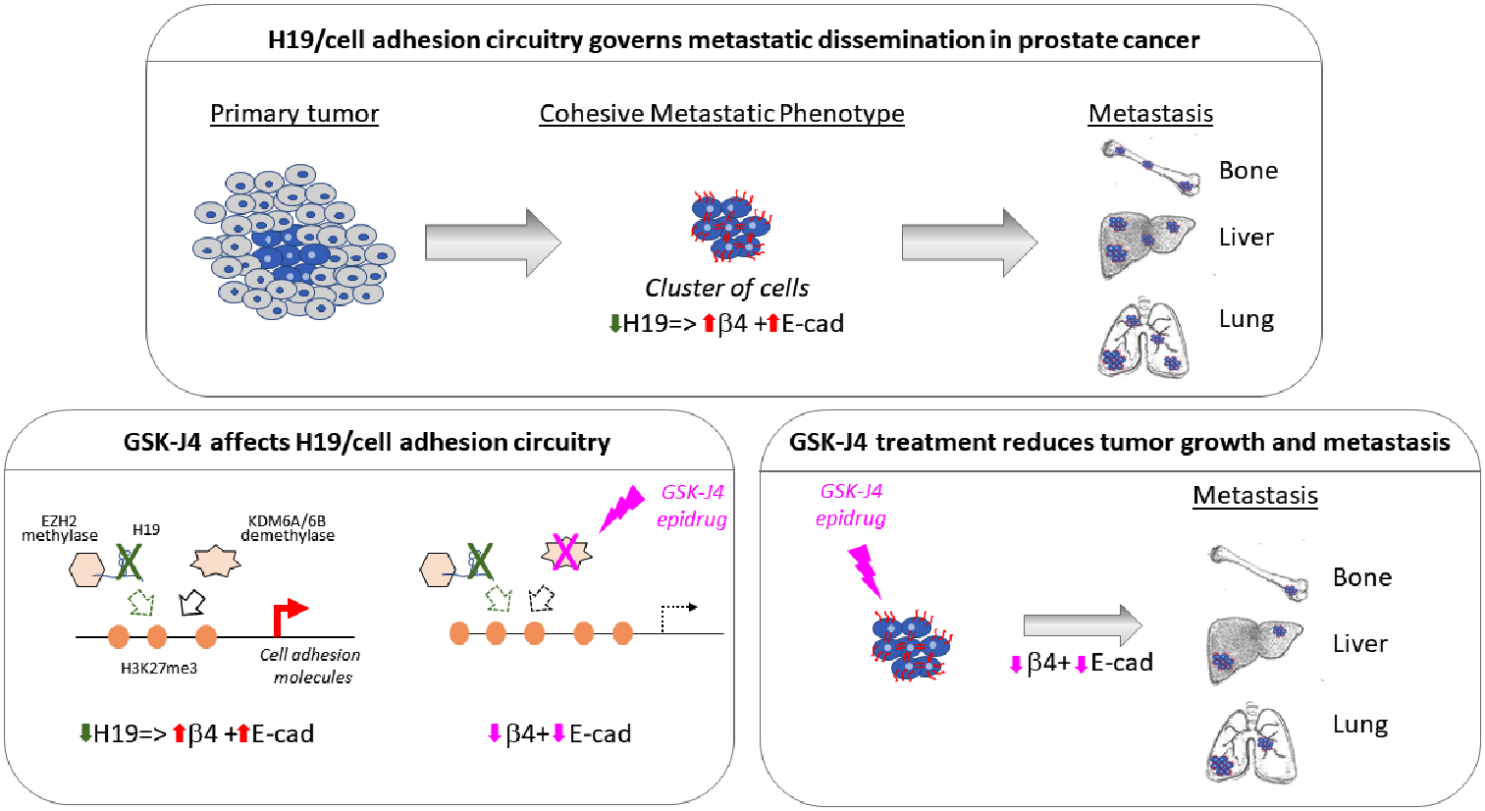
Role of H19/cell adhesion molecules in prostate cancer progression and its epigenetic targeting with reduction of tumor growth and metastasis dissemination

In this study, we recapitulated a picture of poor prognosis PCa by the H19 interefering approach and provided compelling evidence that H19 silencing in PC-3 and 22Rv1 PCa cells causes: (1) induced expression of E-cadherin and β4 integrin; (2) mediated the acquisition of in vitro aggressive phenotype (Fig. 1, S2 and S3), higher tumor growth rate (Fig. 4 and S8) as well as metastatic dissemination in vivo (Figs. 5 and 6 and S9). As the main result, the epigenetic interference of H19/cell adhesion molecules by the cell-permeable histone lysine demethylase inhibitor GSK-J4 restored the basal level of cell adhesion molecules in H19 silenced cells (Fig. 2, S4, and S6) and reduced in vivo tumor growth (Fig. 4) as well as metastasis formation to bone, lung, and liver (Figs. 5 and 6). These findings align with the recent results showing reduced tumor growth after GSK-J4 treatment in subcutaneous xenograft of C42B and LNCaP PCa cells [40, 50]. However, Sanchez and colleagues [50] reported no effect of GSK-J4 on xenografts upon PC-3 and DU145 injection. In this regard, we want to point out that the significant GSK-J4 effect observed on xenograft occurred upon H19 silencing, suggested that GSK-J4 treatment might be effective on aggressive tumour with lower H19 expression by activation of H19/cell adhesion molecules. In addition, this treatment might stress as well the role of H19-mediated epigenetic regulation in our system, essentially associated with the transcriptional control of cell adhesion molecules (Fig. 8). Importantly, we demonstrated the efficacy of GSK-J4 in reducing PCa metastasis, which is consistent with recent observations in osteosarcoma [51]. Furthermore, our results suggest that the effect of GSK-J4 is independent of the androgen/androgen receptor pathway, thus extending its potential as a targeted therapy for castration-resistant prostate cancer.

At the mechanistic level, we investigated the contribution of both KDM6A/UTX and KDM6B/JMJD3 to the up-regulation of E-cadherin and β4 integrin upon H19 silencing (Fig. 3 and S7). In H19 silenced cells, both histone demethylases appear involved in cell adhesion molecules induction as assessed by specific RNA interference, and both are recruited on promoters by ChIP, thus leading to H3K27me3 reduction and transcriptional induction. In this regard, our previous work had already established the regulatory role of JMJD3 in controlling cell adhesion molecules in primary PCa-derived cells [24], and here we have extended this observation to metastatic PCa-derived cells (PC-3 and 22Rv1). About UTX, while it is previously shown that UTX regulates E-cadherin in colon cancer and multiple myeloma cells [52, 53], to the best of our knowledge, this is the first evidence of UTX’s contribution to the transcriptional control of ITGB4, highlighting the influence of the KDM6 family in this process.

To validate the effects of GSK-J4 on different tumor models, we used PCa-derived OSCs that recapitulate tissue architecture representing viable specimens valuable to test pharmacological sensitivity [24, 43, 46, 47]. After OSCs exposure to GSK-J4 for 72 h (Fig. 7), we observed a significant reduction in CDH1 and ITGB4 mRNA levels in a subgroup of samples referred to as “J4 responders”. Importantly, these “J4 responder” OSCs exhibited a significant induction of cell death, indicating that GSK-J4 treatment disrupts the H19/cell adhesion molecules circuitry in ex vivo PCa samples and impairs cell viability. While these observations are limited to a subset of patients, they provide a promising avenue for considering GSK-J4 as a potential therapy, particularly in the subgroup of PCa patients with altered H19/cell adhesion molecules circuitry. Further studies are warranted to explore this aspect, which will be the focus of future investigations.

Overall, this study on in vitro, in vivo, and ex vivo PCa models deepens the molecular basis of metastatic dissemination, highlighting the critical role of H19/cell adhesion molecules circuitry in prostate cancer progression. The effect of the histone lysine demethylase inhibitor GSK-J4 in restoring the basal level of the H19/cell adhesion molecules circuitry revealed its potential as a novel therapeutic approach with implications in PCa management.

## Conclusions

This study opens up to the idea of a promising targeted therapy approach based on the insights gained from molecular characterization. A novel therapeutic strategy emerges by modulating the H19/cell adhesion molecules circuitry using an effective epidrug, validated across a diverse range of experimental prostate cancer models. This targeted therapy holds great potential in addressing the specific molecular alterations associated with prostate cancer progression.

These accomplishments not only expand our knowledge of prostate cancer biology but also suggest a potential approach for personalized treatments and the development of innovative therapeutic interventions tailored to the molecular characteristics of each patient’s tumor.

### Electronic supplementary material

Below is the link to the electronic supplementary material.

Supplementary Material 1

## Data Availability

The datasets used and/or analysed during the current study are available from the corresponding author on reasonable request.

## Abbreviations

PCa: Prostate cancer
ADT: Androgen-deprivation therapies
lncRNAs: Long non coding RNAs
HATs: Histone AcetylTransferases (HATs)
HDACs: Histone DeACetylases
HMTs: Histone Methyltransferases
H3K27me3: Trimethylation of histone 3 lysine 27
CRPC: Castration-resistant prostate cancer
OSCs: Organotypic Slice Cultures
siH19: H19 shRNA interfering
Vector: Scramble vector
oeH19: H19 full length overexpressing
EV: Empty vector
EMT: Epithelial-to-mesenchymal transition
