# Supplementary material for: Targeting of H19/cell adhesion molecules circuitry by GSK-J4 epidrug inhibits metastatic progression in prostate cancer
Source: Cancer Cell Int. 2024 Feb 5;24:56. doi: 10.1186/s12935-024-03231-6 (PMC10845766; doi:10.1186/s12935-024-03231-6)
Supplement: Supplementary file 1 — Supplementary Material 1 [file 12935_2024_3231_MOESM1_ESM.pdf]

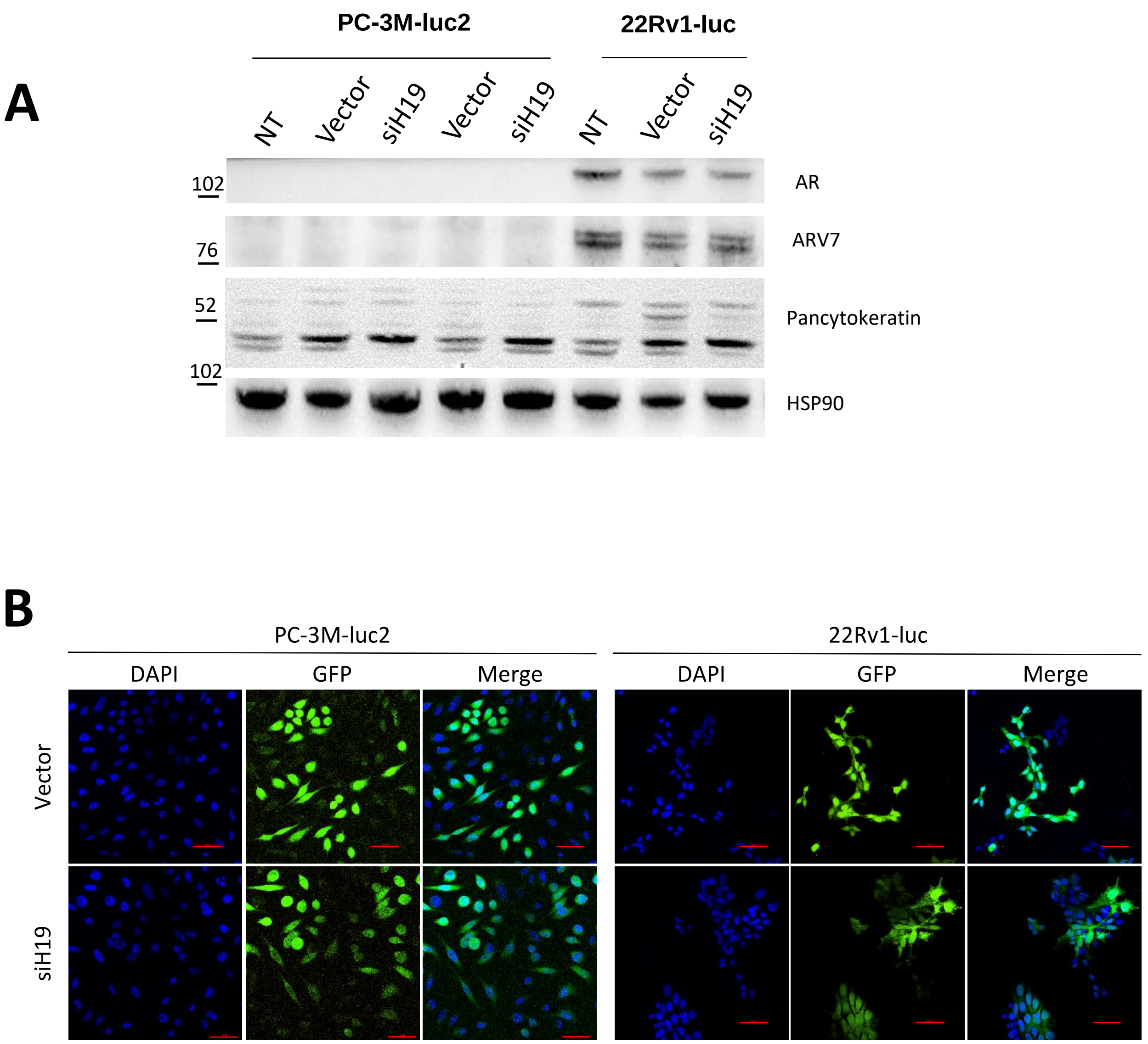

Figure S1. Epithelial Markers and efficiency of lentiviral infection for H19 silencing in PC-3M-luc2 and 22Rv1-luc cells. *A) Analysis by western blot of Androgen Receptor (AR), AR variant (ARv7) and Cytokeratin. HSP90 served as loading control. Molecular weight marker is indicated. B) Efficiency of lentiviral transduction was evaluated by fluorescence up to 90% of GFP-positive cells 5 days after transduction. Cell nuclei were stained by DAPI (blue). Scale bar: 50µm.*

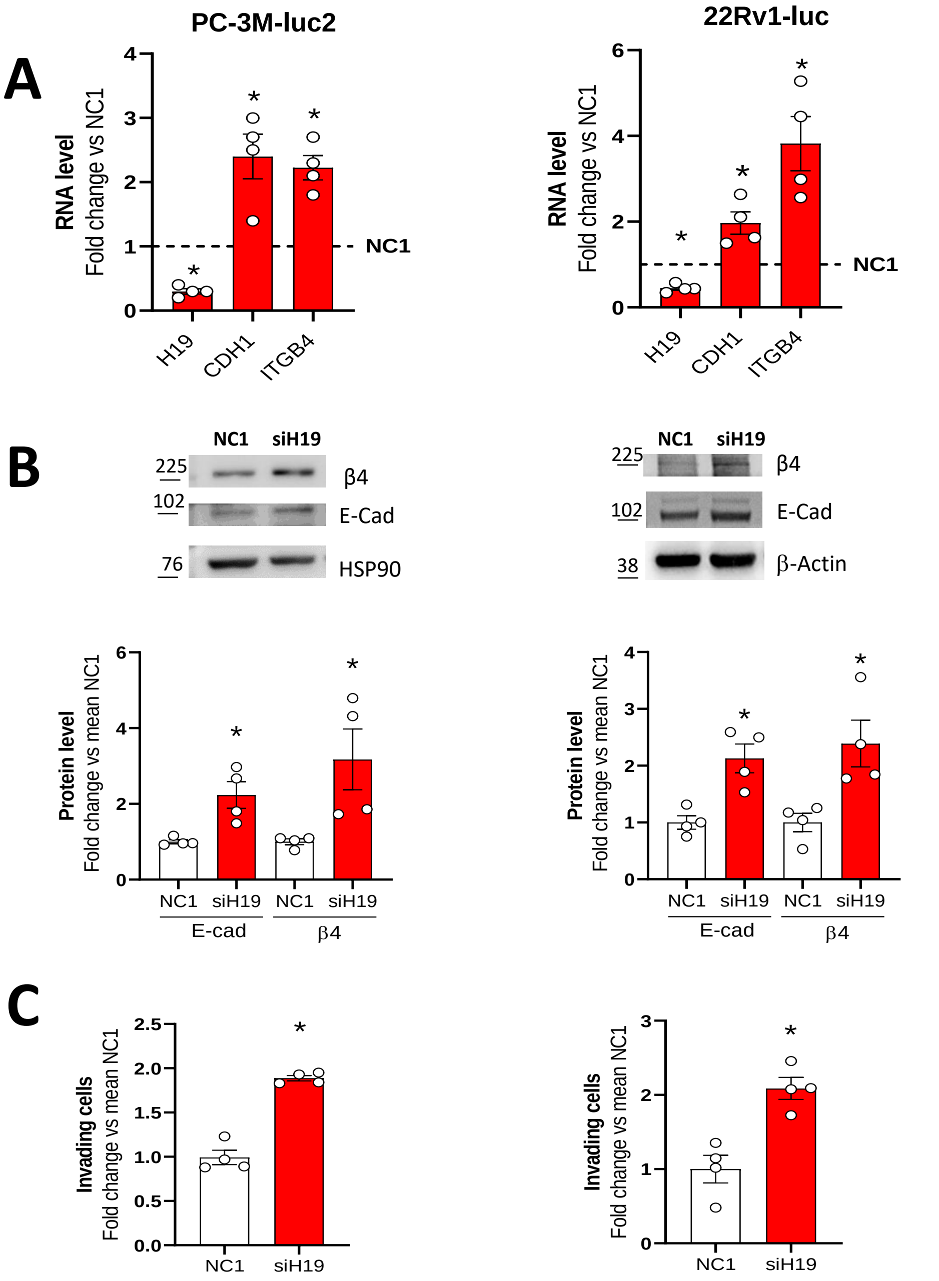

Figure S2. Effects of H19 silencing on cell adhesion molecules expression and metastatic potential in PCa cells. *(A-C) PC-3M-luc2 (left) and 22Rv1-luc (right) cells were transfected with siRNA specific for H19 (siH19) or scramble (NC1) and analysis performed after72h. (A) H19, E-cadherin (CDH1) and β4 integrin (ITGB4) RNA levels were quantified by qPCR. Data, plotted as fold induction vs NC1 (dashed line), represent mean ± SEM of 4 independent experiments (white dots). *P<0.05 vs NC1. (B) Representative E-cadherin (E-cad) and β4 integrin (b4) western blot and densitometry analysis. HSP90 or β-Actin served as control. Molecular weight marker is indicated. Data, plotted as fold change vs. mean NT, represent mean ± SEM of 4 independent experiments (white dots). *P<0.05 vs NC1. (C) Cell invasion by Trans well assay. Lower panel: Data, representing number of invading cell, are plotted as fold change vs. mean NT, represent mean ± SEM of 4 independent experiments (white dots). *P<0.05 vs NC1.*

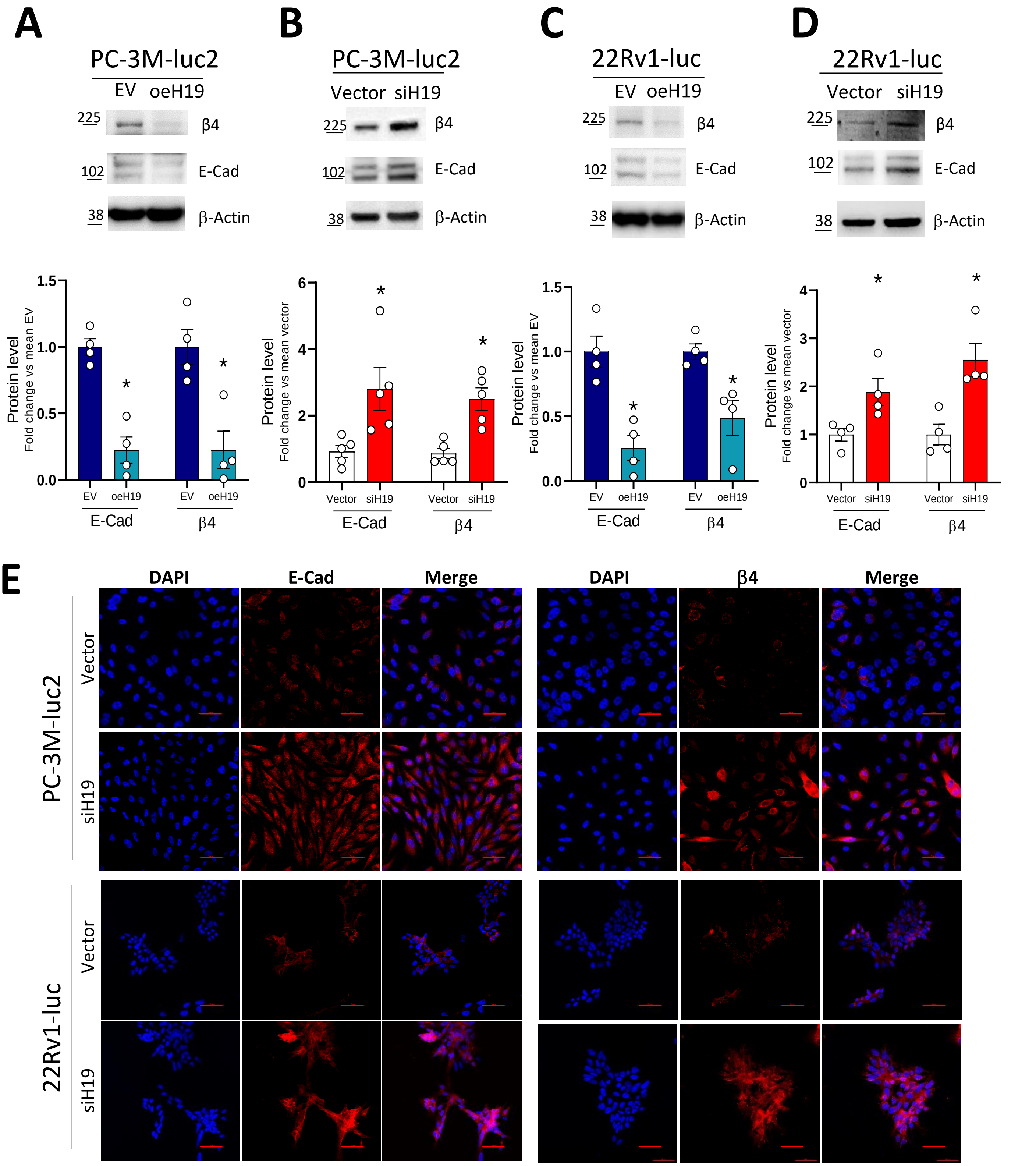

Figure S3. Effect of H19 silencing or over-expression on cell adhesion molecules protein level in PC-3M-luc2 and 22Rv1-luc cells. *(A-D) Representative western blot for E-cadherin and β4 integrin subunit and densitometry analysis in PC-3 and 22Rv1 cells after H19 overexpression (A and C) or H19 silencing (B and D). β-actin was used as a loading control. Data, plotted as fold change vs mean EV or Vector, represent mean ± SEM of 4-5 independent experiments (white dots). Molecular weight marker is indicated*P <0.05 vs Vector. E) E-cadherin (red, left) and b4 integrin (red, right) subcellular localization was evaluated by confocal microscopy in PC-3 and 22Rv1 cells, respectively. Cell nuclei were stained by DAPI (blue) and merged with protein signals. Scale bar: 50 μm.*

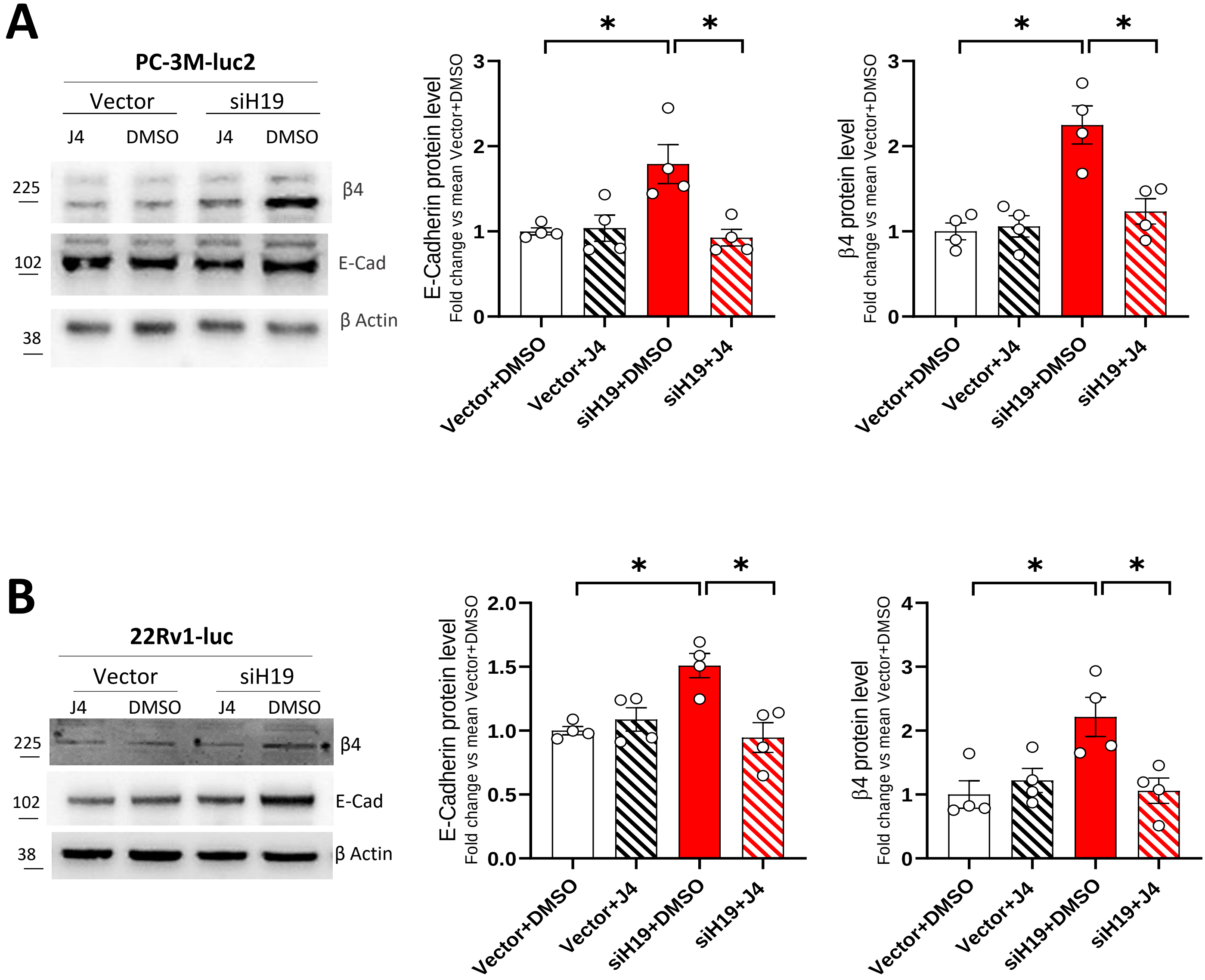

Figure S4. E-cadherin and β4 integrin protein level after GSK-J4 treatment in siH19 cells. *Representative western blot (left) and densitometric analysis (right) for E-cadherin and b4 integrin in PC-3M-luc2 (A) and 22Rv1-luc (B) after 72 hours treatment with GSK-J4 (1uM) or DMSO as control. β-actin was used as a loading control. Molecular weight marker is indicated. Data, plotted as fold change vs mean Vector+DMSO, represent mean ± SEM of 4 independent experiments (white dots*P <0.05.*

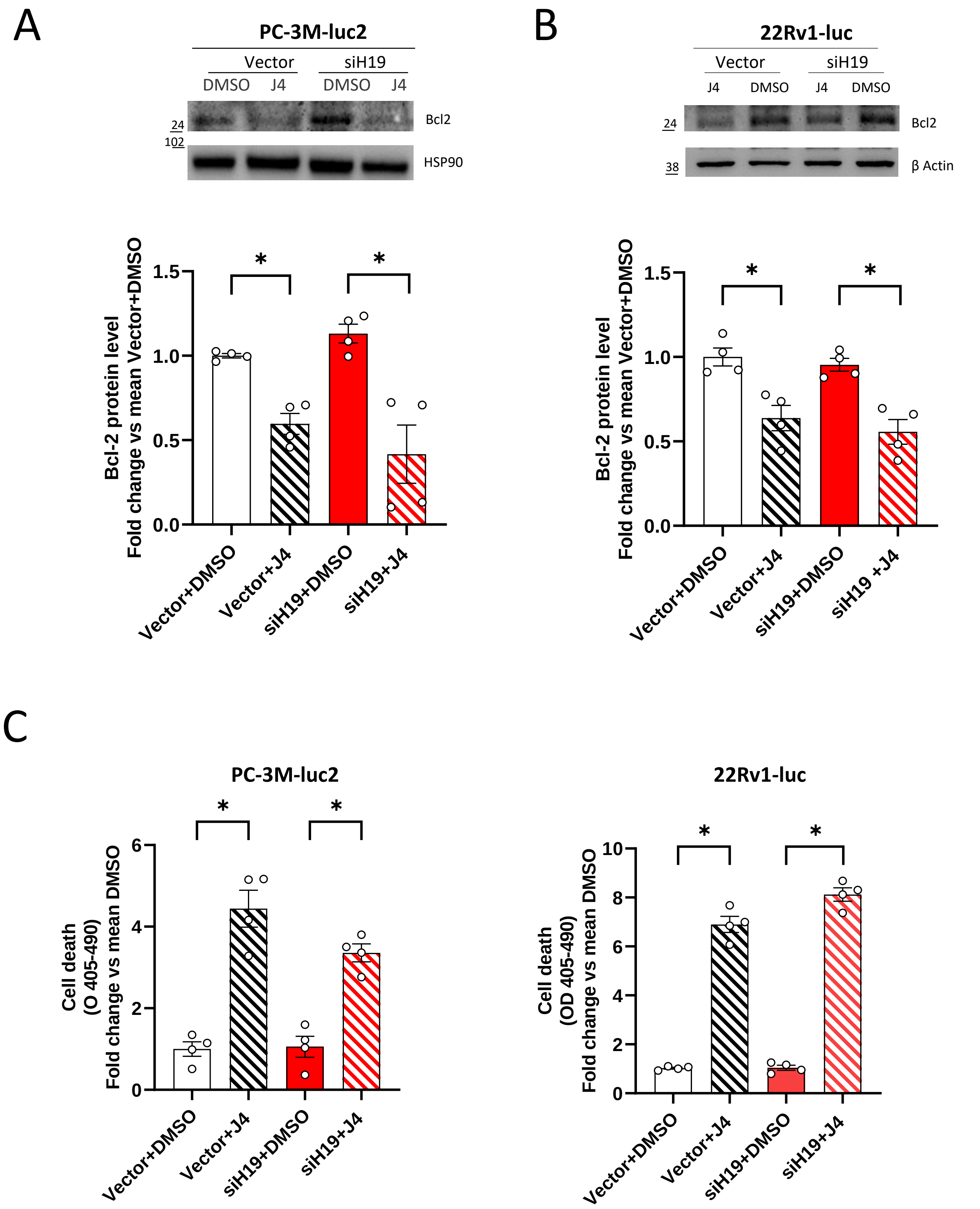

Figure S5. Bcl2 level and Cell death induction after GSK-J4 treatment in siH19 cells. *Representative western blot (left) and densitometric analysis (right) for Bcl2 in PC-3M-luc2 (A) and 22Rv1-luc (B) after 72 hours treatment with GSK-J4 (1uM) or DMSO as control. HSP90 or β-actin was used as a loading control. Molecular weight marker is indicated. Data, plotted as fold change vs mean Vector+DMSO, represent mean ± SEM of 4 independent experiments (white dots).*P <0.05. C) Apoptosis induction upon GSK-J4 treatment evaluated using Cell Death Detection ELISA Kit as described in Methods. Data are expressed as fold change vs. mean DMSO. *P <0.05 J4 vs DMSO.*

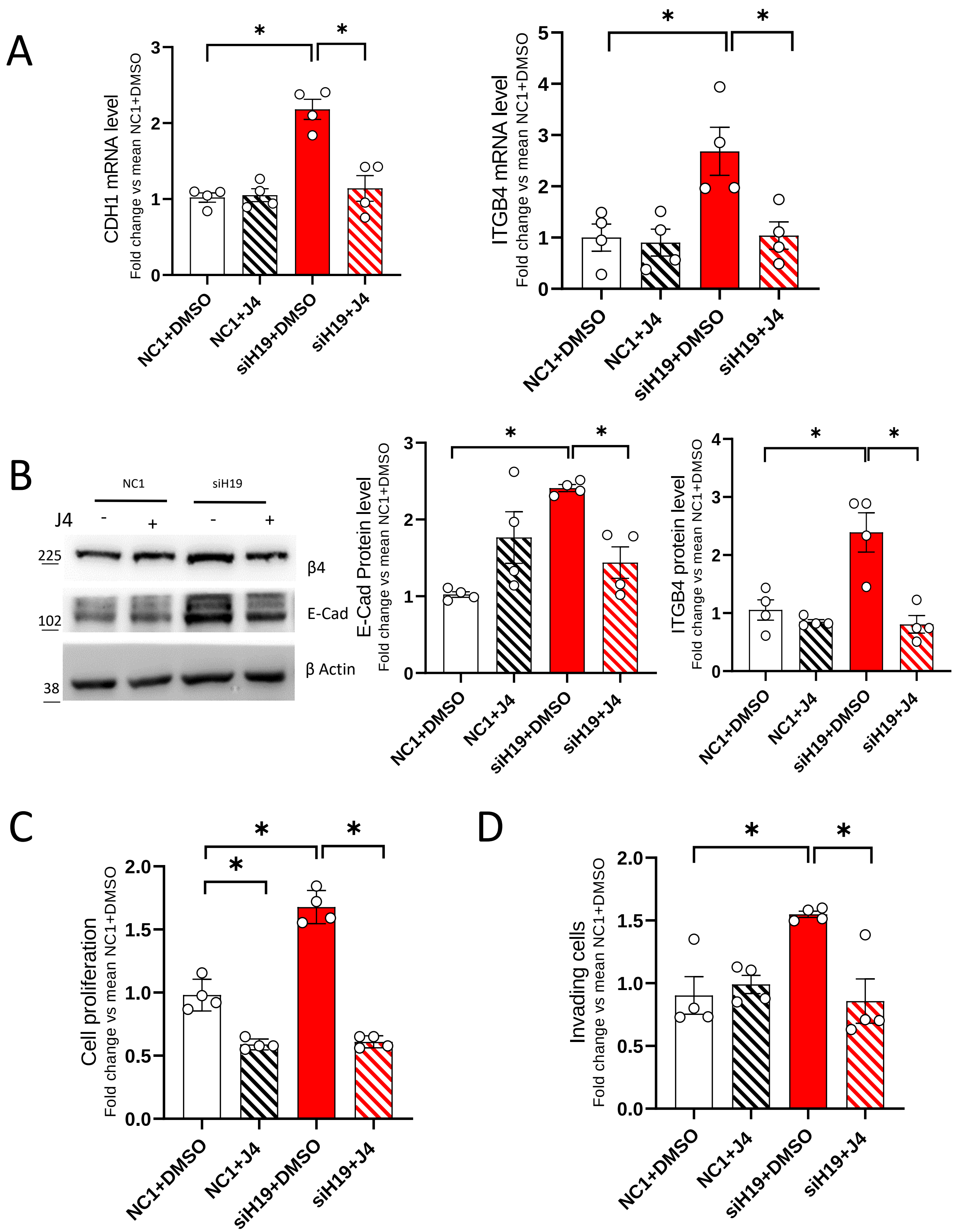

Figure S6. Effect of GSK-J4 in transient H19silenced cells on cell adhesion molecules expression, proliferation and invasiveness in PC-3M-luc2 cells. *Cells were transfected with siRNA specific for H19 or scramble and treated for 72 hours with GSK-J4 (1uM) or DMSO as control. (A) CDH1 and ITGB4 mRNA level assessed by qPCR. (B) Representative western blot and densitometric analysis for E-cadherin and β4 integrin. β-actin was used as a loading control. Molecular weight marker is indicated. (C) Cell proliferation assay. (D) Cell invasion by Trans well assay. Data, plotted as fold change vs. mean NC1+DMSO, represent mean ± SEM of 4 independent experiments (white dots*P <0.05.*

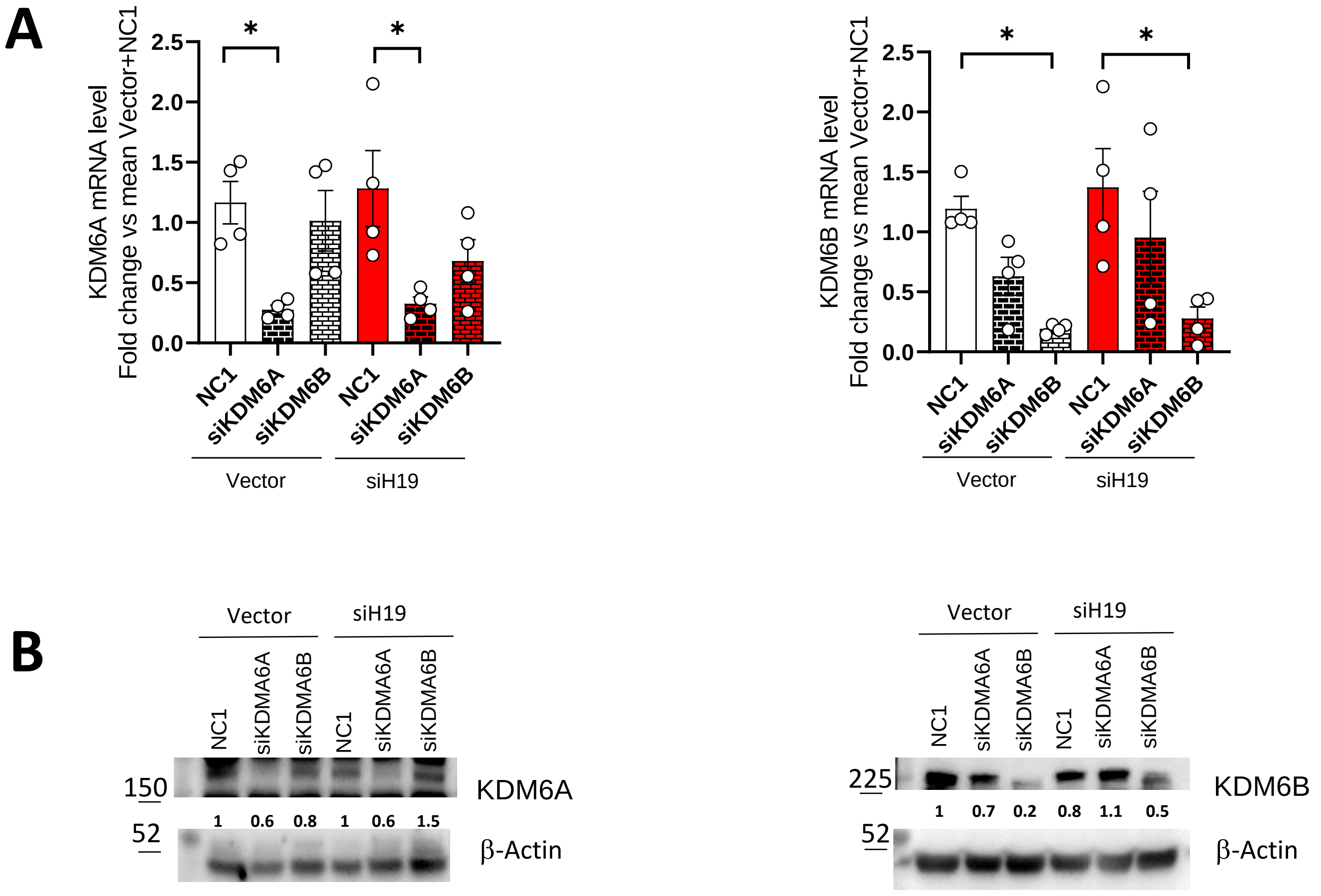

Figure S7. KDM6A and KDM6B silencing in PC-3M-luc2 cells. *SiH19 and Vector cells were transfected with siRNA specific for KDM6A (left panel) and KDM6B (right panel) or scramble (NC1) as control and analysis performed after 72 h. (A) KDM6A and KDM6B mRNA levels were quantified by qPCR. Data are represented as mean ± SEM of fold change vs. mean Vector/NC1 cells of 4 independent experiments (white dots). *P <0.05. (B) Representative western blot for KDM6A (left panel) and KDM6B (right panel). β-actin served as control. Molecular weight marker is indicated. Numbers represent the fold change vs. mean Vector/NC1 normalized to control***.**

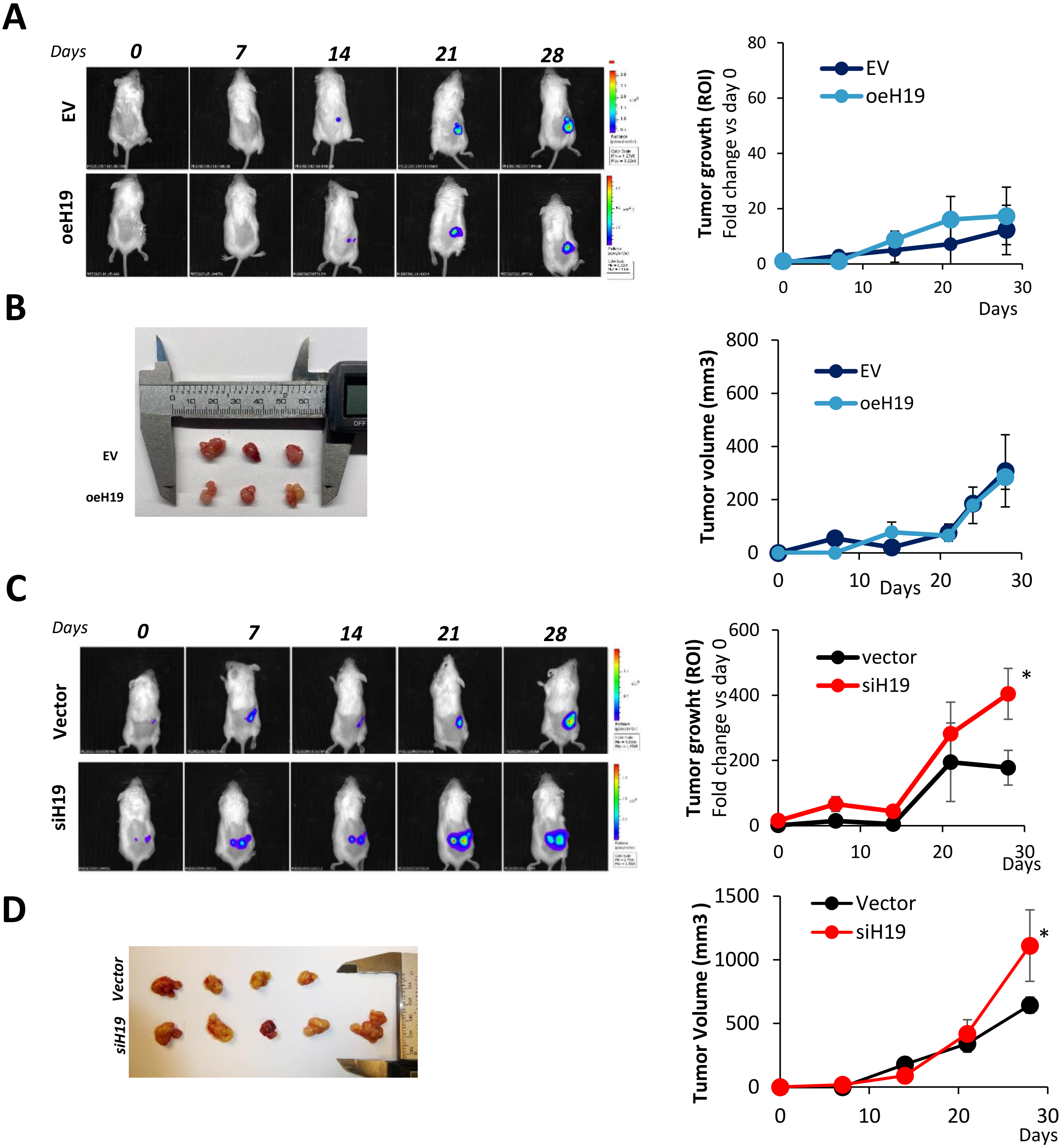

Figure S8. Tumor growth on subcutaneous xenograft mouse model of H19 over-expressing PC-3M-luc2 cells and H19 silenced 22Rv1-luc cells. *(A) Sequential in vivo imaging of tumor growth post subcutaneous injection of H19 over-expressing (oeH19) and Empty Vector (EV) PC-3 cells in NOD/SCID mice. Left: Panels depict a representative mouse from each group. Right: Tumor growth was measured as photons/sec in region of interest (ROI). Data, plotted as fold change vs day 0, represent mean +/- SEM of 6 mice/group. (B) Left: Ex vivo photos of the solid tumors at day of the explant. Right: Tumor volume, as described in legend to Figure 4, represent mean +/- SEM of 6 mice/group. (C) Sequential in vivo imaging of tumor growth post subcutaneous injection of H19 silenced 22Rv1 cells (siH19) and Vector cells in NOD/SCID mice. Left: Panels depict a representative mouse from each group. Right: Tumor growth was measured as photons/sec in region of interest (ROI). Data, plotted as fold change vs day 0, represent mean +/- SEM of 6 mice/group. (D) Left: Ex vivo photos of the solid tumors at day of the explant. Right: Tumor volume, as legend to Figure 4, represent mean +/- SEM of 6 mice/group. *P <0.05.*

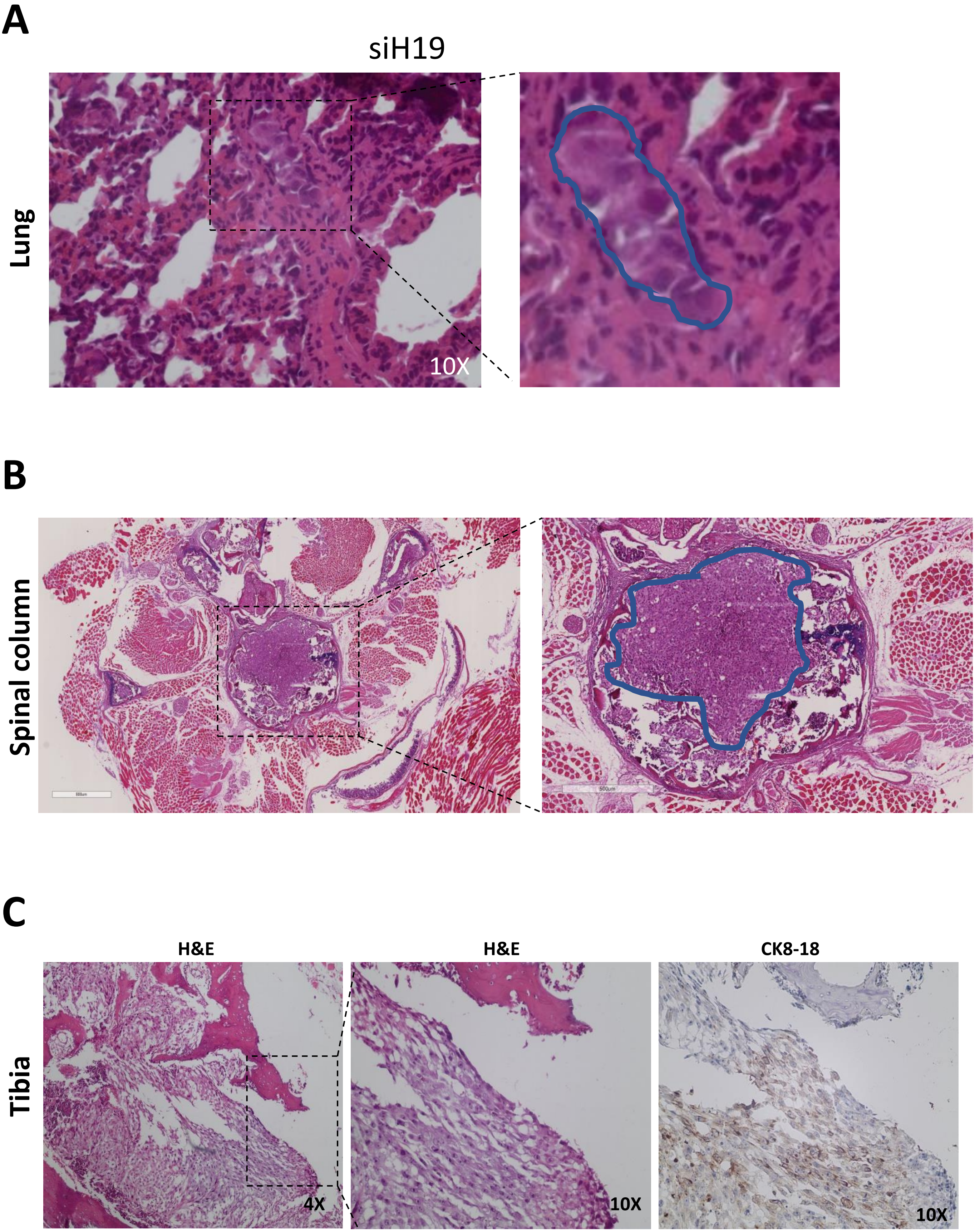

Figure S9. Metastatic dissemination of H19 silenced cells in lung and bone. *Intravenous injection of siH19 and Vector cells in NGS mice as described in legend to Figure 5. A) Representative H&E-stained section of lung with siH19 cells under 10X magnification. Dashed lines indicate at higher magnification in right panel. Right: zoom on metastatic siH19 cluster cells in lung blood vessel (blue line). B) Representative H&E-stained section of column. Scale bar is indicated. Right: Zoom area with metastatic cells indicated by blue line. (C) Representative H&E-stained section of tibia under 4x and 10x magnification. Right: IHC for human cytokeratin CK8-18 under 10x magnification.*

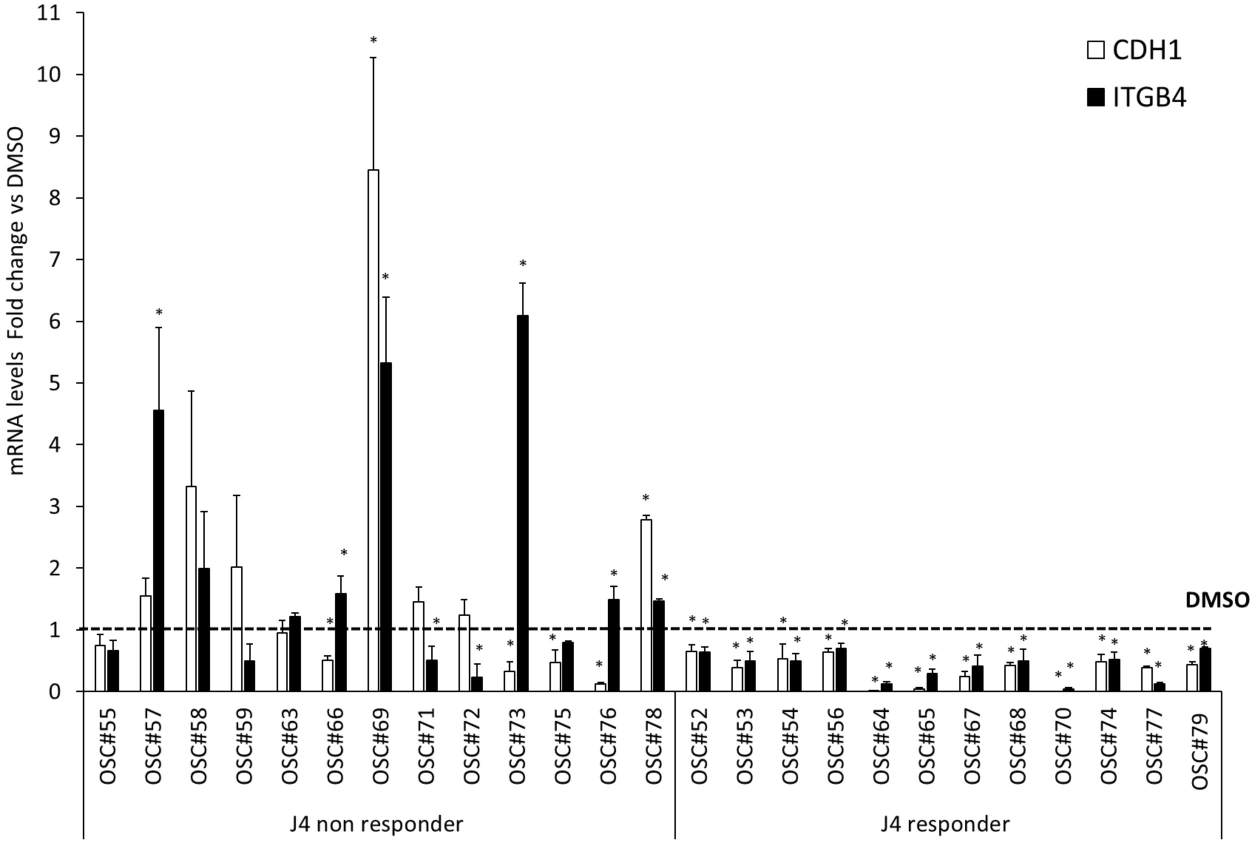

Figure S10. CDH1 and ITGB4 mRNA levels in Organotypic Slices Cultures treated with GSK-J4. *Quantification of CDH1 and ITGB4 transcripts by qRT-PCR in OSCs (n=25) after 72h treatment with GSK-J4 (J4, 5μM) or DMSO as control. OSCs were divided into J4-responder or J4-no responder group according to at least 25% reduction upon treatment of both genes. Data plotted as fold change vs. DMSO (dashed line) represent mean +/-SEM. Statistical significance was determined by Mann-Whitney U test. *P < 0.05 vs. DMSO. B) Quantification of H19 basal level by qRT-PCR in OSCs sub-divided into J4-responder or J4-non responder. White dots indicate a single OSC.*P<0.05*
